# Dynamic inosinome profiles reveal novel patient stratification and gender-specific differences in glioblastoma

**DOI:** 10.1186/s13059-019-1647-x

**Published:** 2019-02-13

**Authors:** Domenico Alessandro Silvestris, Ernesto Picardi, Valeriana Cesarini, Bruno Fosso, Nicolò Mangraviti, Luca Massimi, Maurizio Martini, Graziano Pesole, Franco Locatelli, Angela Gallo

**Affiliations:** 1 RNA Editing Lab., Oncohaematology Department, IRCCS Ospedale Pediatrico “Bambino Gesù”, Viale San Paolo, 15 00146 Rome, Italy; 2 Department of Biosciences, Biotechnology and Biopharmaceutics, University of Bari “A. Moro”, Bari, Italy; 3 Institute of Biomembranes, Bioenergetics and Molecular Biotechnologies (IBIOM), National Research Council, Bari, Italy; 4 Fondazione Policlinico Universitario “A. Gemelli,” IRCCS, UOC Neurochirurgia Infantile, Rome, Italy; 5 Istituto di Neurochirurgia, Università Cattolica del Sacro Cuore, Rome, Italy; 6 Fondazione Policlinico Universitario “A. Gemelli”, IRCCS, UOC Anatomia Patologica, Rome, Italy; 7 Istituto di Anatomia Patologica, Università Cattolica del Sacro Cuore, Rome, Italy; 8 Department of Pediatrics, “La Sapienza” University, Rome, Italy

**Keywords:** RNA editing, ADAR, GBM, RNA-Seq, COG3

## Abstract

**Background:**

Adenosine-to-inosine (A-to-I) RNA editing is an essential post-transcriptional mechanism mediated by ADAR enzymes that have been recently associated with cancer.

**Results:**

Here, we characterize the inosinome signature in normal brain and de novo glioblastoma (GBM) using new metrics that re-stratify GBM patients according to their editing profiles and indicate this post-transcriptional event as a possible molecular mechanism for sexual dimorphism in GBM. We find that over 85% of de novo GBMs carry a deletion involving the genomic locus of *ADAR3*, which is specifically expressed in the brain. By analyzing RNA editing and patient outcomes, an intriguing gender-dependent link appears, with high editing of *Alus* shown to be beneficial only in male patients. We propose an inosinome-based molecular stratification of GBM patients that identifies two different GBM subgroups, INO-1 and INO-2, which can identify novel high-risk gender-specific patient groups for which more aggressive treatments may be necessary.

**Conclusions:**

Our data provide a detailed picture of RNA editing landscape in normal brain and GBM, exploring A-to-I RNA editing regulation, disclosing unexpected editing implications for GBM patient stratification and identification of gender-dependent high-risk patients, and suggesting COG3 I/V as an eligible site for future personalized targeted gene therapy.

**Electronic supplementary material:**

The online version of this article (10.1186/s13059-019-1647-x) contains supplementary material, which is available to authorized users.

## Background

Genomic instability and increased DNA mutation frequency provide selective advantages for clonal multiplication of cancerous cells. More than 100 oncogenes have been identified so far, but only a small subset has been consistently classified as cancer driver genes [1, 2]. Altogether, alterations affecting genes able to modulate RNA expression, cell differentiation, and recoding genomic messages are recognized as important factors for cancer progression. Interestingly, an essential post-transcriptional mechanism, A-to-I RNA editing, is able to modulate all the above molecular pathways, and for this reason, increasing attention has been paid to this mechanism in cancer field [3, 4].

RNA editing has a critical role in superimposing novel/additional information to the genetically hard-wired transcriptome [5]. The most common type of RNA editing in humans consists in A-to-I nucleotide conversion within double-stranded RNA (dsRNA) molecules catalyzed by the adenosine deaminases that act on the dsRNA (ADARs) family of enzymes. ADAR1 (also known as ADAR) and ADAR2 (also known as ADARB1) are active and ubiquitously expressed enzymes [5], while ADAR3 (also known as ADARB2) is inactive and mainly expressed in the brain, where it seems to regulate the editing activity of the other two ADARs [6, 7]. Inosines are recognized as guanosines by splicing and translational machineries ultimately leading to diversification of both the transcriptome and proteome landscapes [8].

So far, it has been estimated that over 4.7 million editing sites exist in the human transcriptome, involving both coding and non-coding RNAs [8–10]. ADARs are essential enzymes in mammals [11, 12]; however, it remains to be defined which editing sites are necessary for cell homeostasis and survival or important in cancer [13–15].

Parallel advances in computational methods and high-throughput RNA sequencing have supported the massive identification of RNA editing sites, demonstrating that the vast majority of editing in humans occurs within the primate-specific *Alu*-inverted repeat elements, due to their tendency to form dsRNA secondary structures, substrates recognized by ADAR enzymes [16, 17]. Virtually, all adenosines within *Alu* repeats are edited, mostly to a low degree (< 1%) [16]; however, specific sites (such as non-repetitive or recoding sites) can be edited at high level, with *GRIA2* Q/R site being an example of a highly edited site in the brain (~ 100% editing) [11].

Recent studies, mainly conducted thanks to The Cancer Genome Atlas (TCGA) project, have characterized the RNA editing landscape of various cancer types in a systematic way [14, 18, 19]. These studies, searching for common editing features among cancer types, revealed many altered A-to-I RNA editing events in tumor samples relative to the normal tissues.

Herein, we specifically focused on de novo*/*primary glioblastoma (GBM), normal brain, and astrocytes in a robust and integrated study analyzing these samples in detail at post-transcriptional level also combining genomic/transcriptional data, thus providing an alternative approach to stratify GBM patients (INO-1 and INO-2) and revealing novel findings potentially critical for future personalized therapies. Of note, de novo GBM is the most common and deadly primary brain tumor in humans developing from glial cells, for which more effective therapeutic interventions are urgently needed [20]. The vast majority of GBMs (~ 90%) develop rapidly as observed in de novo GBMs, while the less frequent, secondary GBMs progress from lower-grade astrocytomas. In order to elucidate de novo GBM-specific RNA editing signature, we analyzed RNA-Seq from 145 primary GBMs (TCGA dataset) compared to 132 RNA-Seq (GTEx project) from normal brain cortex and to 12 pools of primary astrocytes which represent the majority of glial cells in the brain. Additionally, we also confirmed our major results in a second independent data set of glioblastomas (CGGA).

For the first time, our study characterizes GBM inosinome in a comprehensive and transcriptome-wide manner, disclosing a strong and peculiar perturbation of inosinome landscape in GBM compared to normal brain never described before and involving all the three ADAR proteins as tested in two independent de novo GBM cohorts of patients (TCGA and CGGA). Most importantly, we showed that the editing signature can stratify GBM patients and identify gender-dependent high-risk patients never reported before. Altogether, our findings provide novel insights into A-to-I RNA editing regulation and GBM pathogenesis.

## Results

### Comprehensive A-to-I RNA editing analysis showed a consistent decrease in editing activity in de novo GBM compared with both normal brain and primary astrocytes

**Fig. 1:**
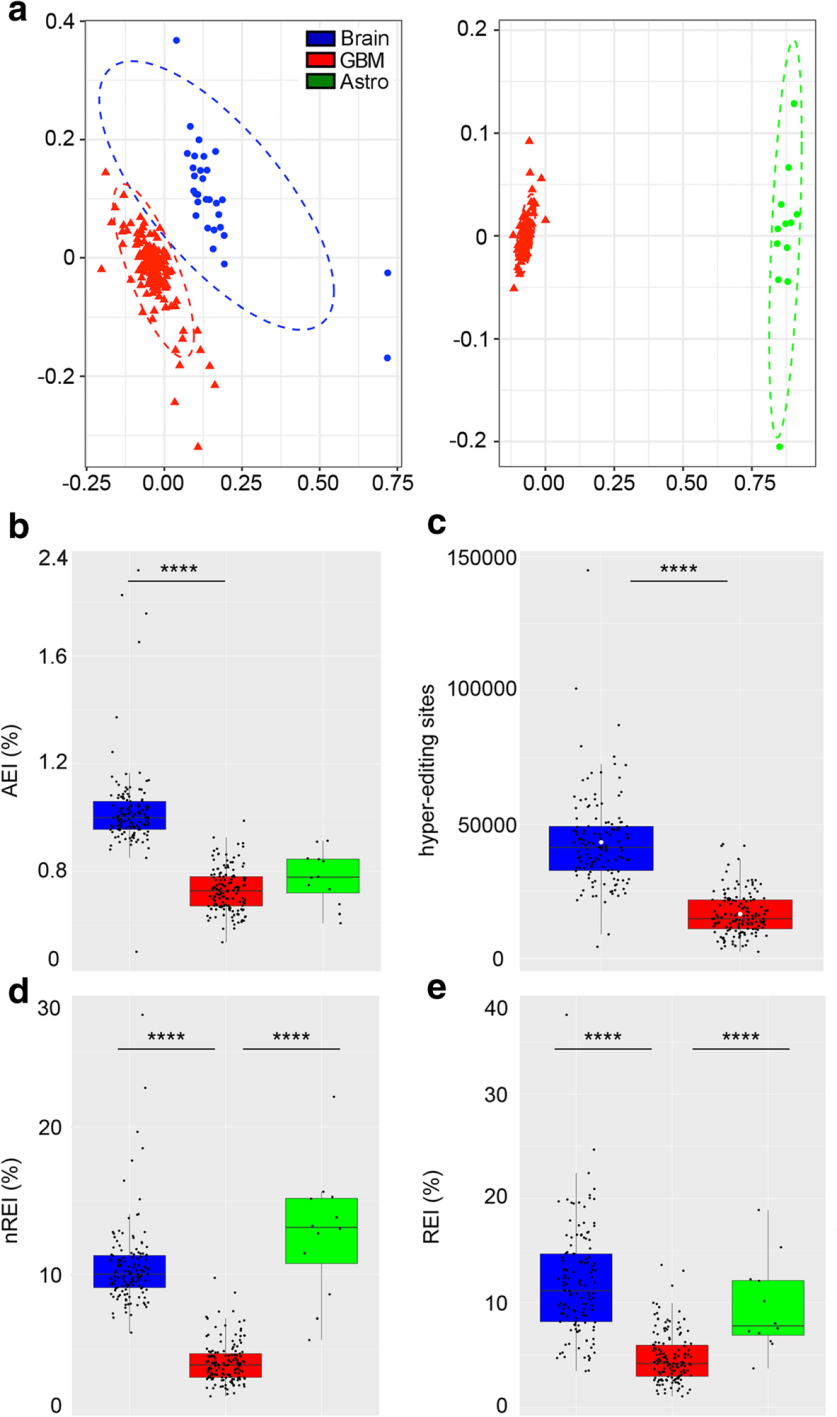
Inosinome signature in de novo GBM compared to the normal brain and astrocytes. **a** Two-dimensional non-metric multi-dimensional scaling (N-MDS) ordination plots of GBM (145 samples, shown in red), cerebral cortex (132 samples, shown in blue), and normal astrocytes (12 different pools, shown in green). **b** *Alu* editing index (AEI) distributions (box plot, median). **c** Distributions of hyper-editing sites (box plot, median and mean values indicated as a black bar and white dots, respectively). **d** Non-repetitive editing index (nREI) value distributions (box plot, median). **e** Recoding editing index (REI) values distributions (box plot, median). Two-tailed Mann-Whitney *U* test was applied. ****p ≤ 0.0001

We started by studying the similarities and distances among de novo GBMs (145 samples), normal brain tissues (132 samples), and primary astrocytes (12 independent pools) based on editing profiles by applying a non-metric multi-dimensional scaling (N-MDS) method based on Spearman’s rank correlation coefficients. We showed that de novo GBM inosinomes always clustered as a distinct and independent group compared to either the brain cortex or astrocyte samples, being more similar to normal brain tissues than astrocytes (Fig. 1a).

Global editing activity mainly concentrates on *Alu* sequences; therefore, we initially focused on quantifying editing within *Alus* in order to explore the global editing pattern among samples. We calculated the *Alu* editing index (AEI) and then compared the different distributions. Of note, the AEI corresponds to the weighted average editing level across all the expressed *Alu* sequences [21]. Applying this metric, we found a strong editing decrease in GBMs compared to normal brain samples (median values GBM = 0.73% and brain cortex = 1%, *p* < 0.0001) (Fig. 1b; Additional file 1: Table S1). We then extended our analysis to the sites located in hyper-edited regions (i.e., repetitive RNA portions with clusters of multiple edited adenosines) that are undetectable by standard alignment methods [22], finding that cancer samples are significantly under-edited also in these regions (Fig. 1c; Additional file 1: Table S1). Hyper-editing values were not tested in astrocyte pools due to the low number of potentially supporting reads per sample required for this specific analysis.

While most editing sites are located within *Alu-*inverted repeats, several editing events occur in non-repetitive regions of RNAs, with some of them leading to amino acid substitutions (recoding sites) often present in key neuronal gene transcripts [23]. The latter sites are potentially highly informative as they are more likely to have a functional role in cancer. In order to quantify the overall fluctuations of editing levels at those sites, we introduced a novel, ad hoc, metrics for the unbiased measure of global editing at non-repetitive and recoding sites, generating two indexes that we named “non-repetitive editing index” (nREI) and “recoding editing index” (REI). These indexes represent the weighted average editing levels at all known non-repetitive and recoding sites, respectively.

Applying these new metrics, we found that overall editing, also at non-repetitive and recoding sites, was significantly and strongly decreased in GBMs compared to either the brain cortex or astrocytes (nREI median values GBM = 3.94%, cortex = 10.03%, astrocytes pools = 13.2%; REI median values GBM = 4.18%, cortex = 11.15%, astrocytes = 7.82%, *p* < 0.0001) (Fig. 1d, e; Additional file 1: Table S1).

An additional RNA-Seq data generated from an independent non-overlapping de novo GBMs cohort, the Chinese Glioma Genome Atlas (CGGA) [24], was also utilized to validate our findings. Indeed, we report that in both the de novo GBM cohorts (TCGA and CGGA), the editing levels, as measured by the different indexes (AEI, nREI, and REI), significantly decreased in tumor compared to the normal brain (Additional file 2: Figure S1a).

Taken together, our results demonstrate that de novo GBMs have a distinct inosinome profile compared to both normal brain and astrocytes (the most abundant fraction of glial cell types in the brain) characterized by a strong and global editing decrease affecting different positions: *Alus*, hyper-edited, non-repetitive, and recoding sites.

### Changes in RNA editing patterns at recoding sites in GBMs relative to normal samples: re-shaping the RNA editing signature at recoding sites

Considering the possible relevant importance of recoding editing sites in cancer [25], we specifically focused on these sites. We systematically searched for significant alterations of editing in recoding sites in the brain and GBM finding that among the 397 informative recoding positions (supported by at least 10 reads/site and with ≥ 1% editing), only 178 sites were found significantly differently edited in GBM compared to the normal brain (Additional file 3: Table S2, two-tailed Mann-Whitney *U* test followed by Benjamin-Hochberg multiple test correction). Among these, 89 sites were localized in non-repetitive regions (identified in Additional file 3: Table S2 as NONREP), 79 sites in *Alu* repeats (identified as ALU), and only 10 sites in repetitive (identified as REP) non-*Alu* regions (i.e., LINE, non-*Alu* SINEs) (Additional file 3: Table S2).

We report that > 93% (166/178) of the identified recoding sites were significantly under-edited in GBM, with only 12/178 sites showing an increased editing level (Additional file 3: Table S2). Additionally, among the 12 over-edited sites in GBM, 10 sites showed a slight editing increase in GBM ranging from 1 to 5% Δ medians (Additional file 3: Table S2) and only 2 sites displayed an editing increase > 15% Δ medians in GBMs compared to the normal brain: *COG3* I/V and *CADPS* E/G (both with Δ medians = 17%) (Additional file 3: Table S2).

**Fig. 2:**
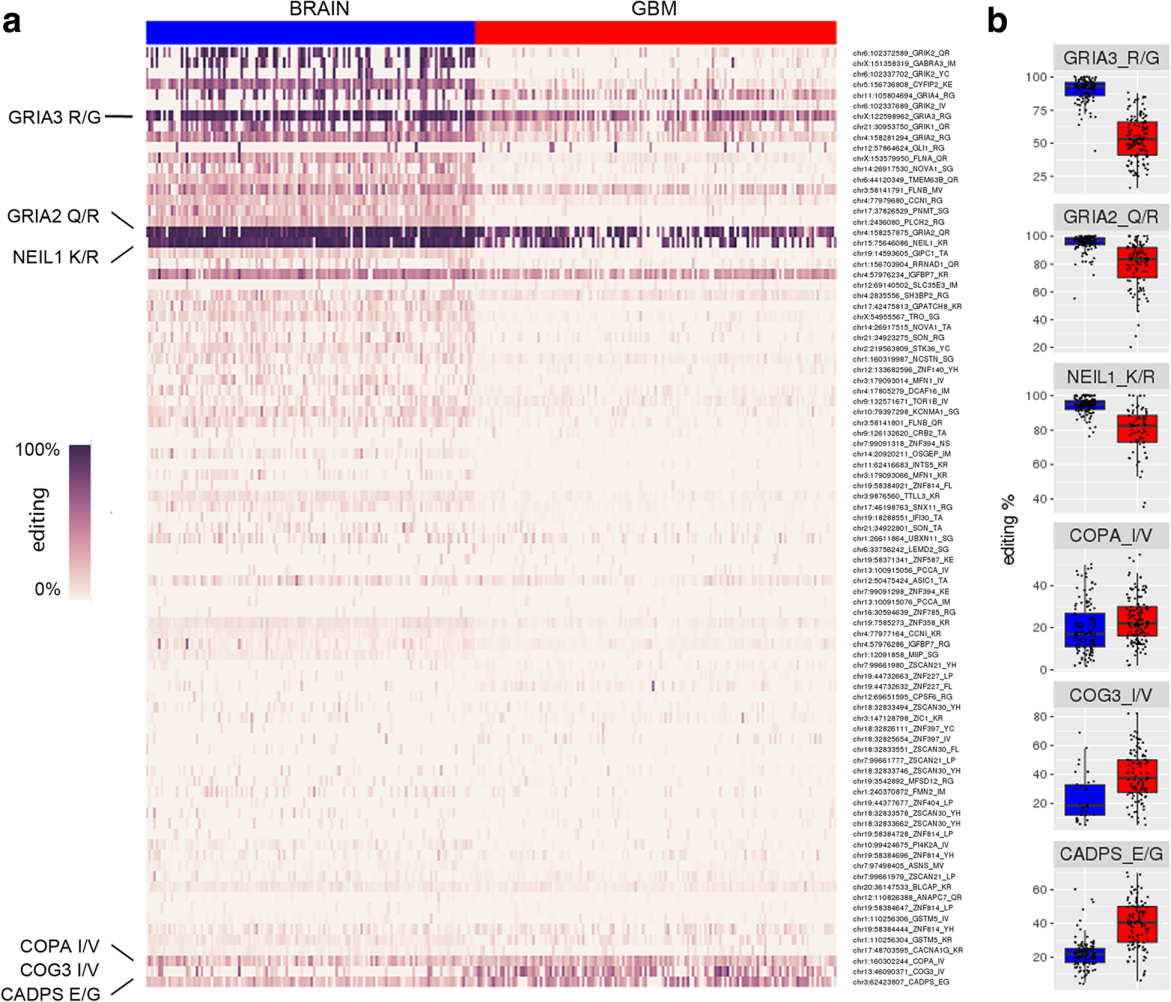
Editing fluctuation in de novo GBM and normal brain at coding sites. **a** Heatmap of RNA editing levels at recoding sites in GBM compared to the normal cerebral cortex. Each column represents one of the 145 de novo GBMs (TCGA) and 132 brain cortex (GTEx) samples, rows show the 89 differentially edited non-repetitive recoding sites. Only the statistically significant sites (two-tailed Mann-Whitney *U* test, with Benjamini-Hochberg-corrected *p* value ≤ 0.05) are shown. The heatmap was generated with Pandas and Seaborn Python libraries, and the list of the editing sites was also reported in the same order in Additional file 3: Table S2. **b** Box plots representing editing frequencies (%) distributions in GBM and brain cortex at selected sites (two-tailed Mann-Whitney test, with Benjamini-Hochberg correction): *GRIA3* R/*G q* value = 6.44E−33, *GRIA2* Q/R *q* value = 8.63E−20, *NEIL1* K/R *q* value = 1.01E−16, *COPA* I/V *q* value = 0.0022, *COG3* I/V *q* value = 0.0002, and *CADPS* E/G *q* value = 2.58E−18. In red GBM and in blue normal brain cortex samples are shown. Medians are indicated by black bars

Editing levels at the 89 recoding sites in non-repetitive regions were shown as a heatmap with a clear and generalized decrease of editing frequency in GBMs compared to the normal brains (Fig. 2a). Few positions (such as *GRIA3* R/G, *GRIA2* Q/R, and *NEIL1* K/R sites) highly edited in the brain resisted better than other sites to the generalized editing decrease phenomenon/scheme, retaining editing at levels similar to those observed in the normal brain (Fig. 2a, b; Additional file 3: Table S2). The few recoding sites being more edited in GBM (i.e., *COPA* I/V, *COG3* I/V, and *CADPS* E/G) were marked in the heatmap (Fig. 2a, b; Additional file 3: Table S2).

We validated by Sanger sequencing the editing level at the *COPA* I/V, *COG3* I/V, and *CADPS* E/G sites in de novo GBMs and the normal brain cortex (Additional file 2: Figure S2a). Additionally, we reported that these sites were all edited by ADAR2, as tested in HEK293T and U87-MG GBM cell lines (Additional file 2: Figure S2b).

Editing at the 89 recoding sites in non-repetitive regions encompassed 65 different transcripts. We found that 83% of the differentially edited transcripts were also aberrantly expressed in GBMs, with 63% of them (41/65) being both under-edited and down-expressed in de novo GBM (Additional file 2: Figure S3).

Overall, our data showed that transcripts carrying recoding editing sites were generally under-edited and under-expressed in de novo GBM compared to normal brain tissue. However, a few specific recoding sites displayed higher editing levels in tumor tissues, suggestive of a possible pro-tumoral role in GBM.

### Functional effects of the *COG3* I/V editing site in GBM

**Fig. 3:**
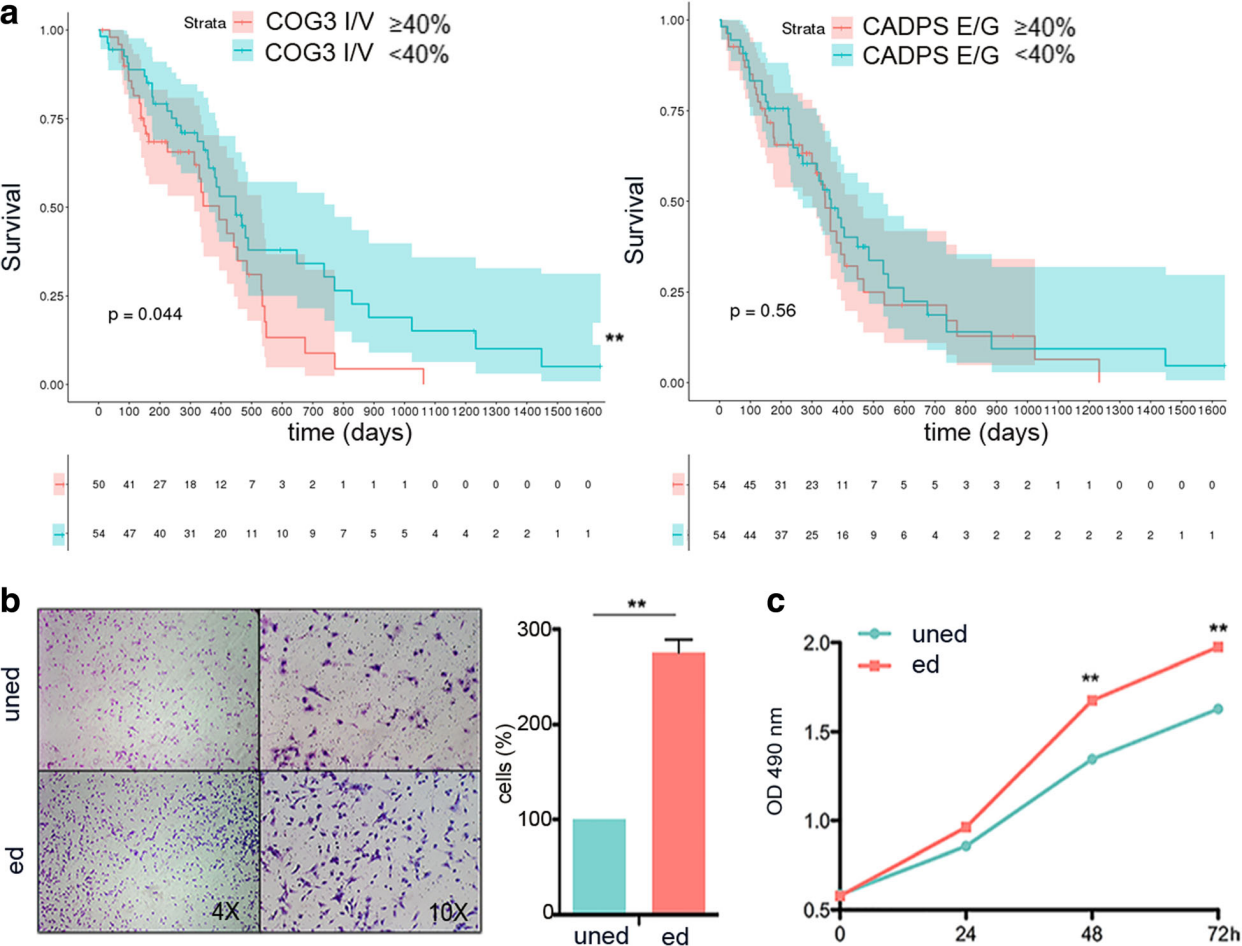
Clinical relevance of editing at *COG3* I/V recoding site. **a** Kaplan-Meier curves representing the survival probability of GBM patients stratified by *COG3* I/V (104 samples) and *CADPS* E/G (108 samples) editing levels respectively. Log-rank test, *COG3 p* value = 0.044, *CADPS p* value = 0.56. The red line represents high editing frequency (≥ 40%), the green line represents low editing frequency (< 40%). **b** Migration assay of glioblastoma cells (A172) expressing unedited *COG3* (uned) and edited (ed) *COG3* was performed 24 h post-seeding. Representative photographs of migrated cells are shown (× 4 and × 10 magnifications). Migrated cells were stained with Diff-Quick and counted. Histograms show the migration ability of ed. *COG3* expressing cells relative to the uned *COG3* (fold increase ± SD, *n* = 3) **p ≤ 0.01 (two-sided *t* test). **c** Proliferation (MTS assay) of glioblastoma cells (A172) infected with unedited or edited *COG3* (mean ± SD, *n* = 3) **p ≤ 0.01 (two-sided *t* test)

A few recoding sites are highly edited in GBM compared to the normal brain with the top positions being *COG3* I/V and *CADPS* E/G (with > 15% editing increase in cancer) (Fig. 2 and Additional file 3: Table S2). We first tested if editing at these sites was associated with patient overall survival (OS), finding that only the *COG3* I/V site, and not the *CADPS* E/G site, was significantly associated with patients’ OS (*p* < 0.044) (Fig. 3a). Specifically, a higher editing level (≥ 40%) at this site is associated with a worse prognosis, suggesting *COG3* I/V over-editing as a possible pro-tumoral event (considering 20% median editing in normal brain, see Additional file 3: Table S2).

We then investigated the possible biological functional role played by *COG3* I/V editing in GBM. We expressed *COG3* in its unedited (uned) or edited (ed) versions in three different glioblastoma cell lines (A172, U87-MG, and U118-MG), and cell proliferation and migration were tested (Fig. 3 and Additional file 2: Figure S4). We found that only the edited *COG3* (edited at I/V site) significantly enhanced the invasive/proliferative behavior in all these three cell lines compared to the unedited counterpart (Fig. 3 and Additional file 2: Figure S4).

Altogether, our data indicated that over-editing (≥ 40%) at the *COG3* I/V site plays a critical pro-tumoral role in GBM and correlates with a worse prognosis in GBM patients.

### *ADAR* expression is altered in GBMs and correlated with editing indexes, age, and gender in the normal brain

**Fig. 4:**
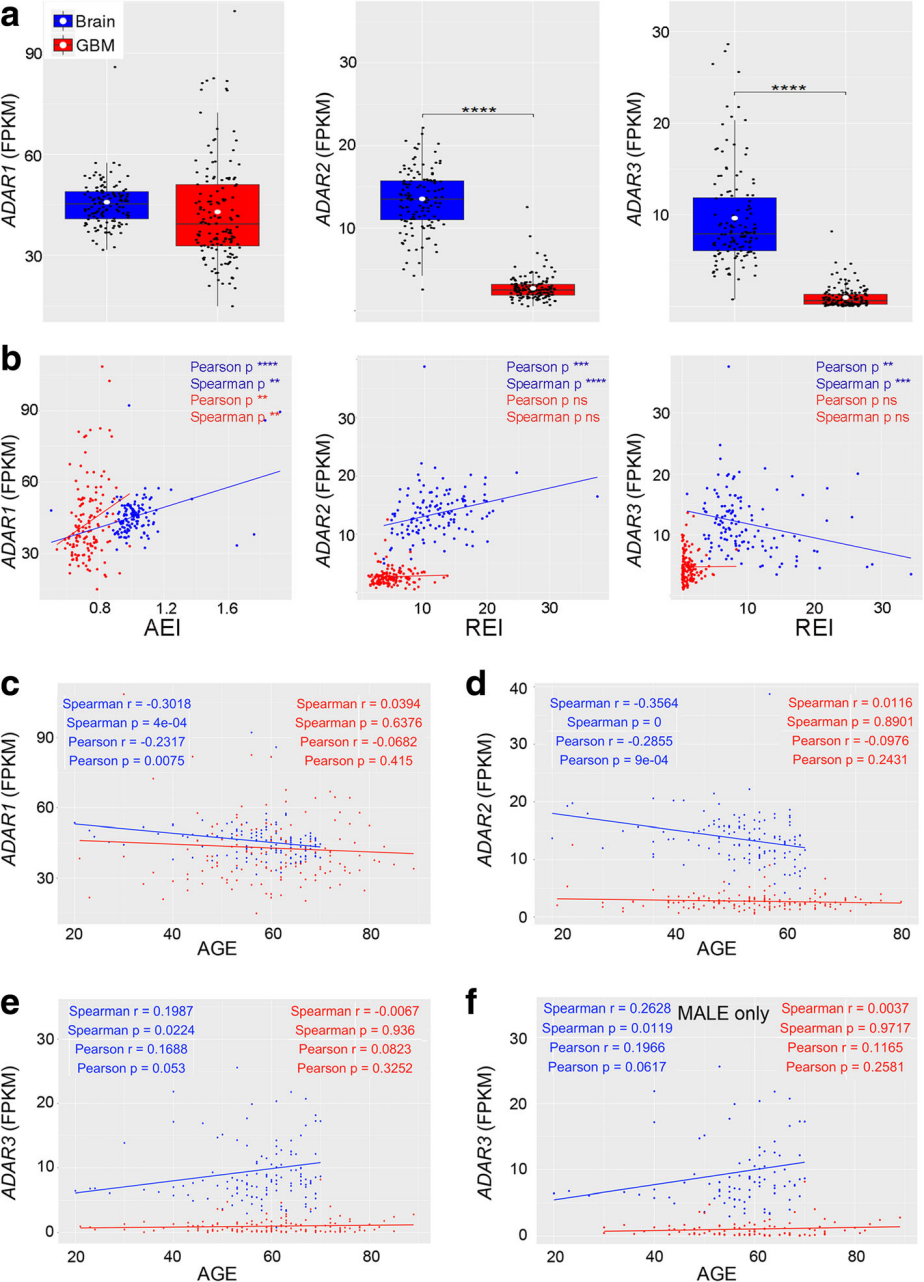
*ADAR* expression correlates with editing indexes, age, and gender. **a** *ADAR1*, *ADAR2*, and *ADAR3* expression levels were calculated by using Cufflinks (FPKM distributions), ****p ≤ 0.0001. **b** Correlations between *ADAR* expressions and editing indexes (AEI and REI) in TCGA GBM cohorts and the normal brain. **c**–**e** Correlations between *ADAR* expression (FPKM) and age (in blue healthy individuals in red GBM patients). **f** Correlation between *ADAR3* expression (FPKM) and male’s age. **p ≤ 0.01, ***p ≤ 0.001, ****p ≤ 0.0001

Editing fluctuations/alterations may be accounted to the deregulation of RNA editing enzyme (ADAR) expression; therefore, we estimated *ADAR* levels in the normal brain and de novo GBMs by using the Cuffquant/Cuffdiff2 pipeline. We found that *ADAR1* is less expressed, although not significantly, in de novo GBMs compared to controls (Fig. 4a). Of note, by applying the Kolgomorov-Smirnov test, which quantifies the distance between distributions, *ADAR1* FPKM values in the brain cortex and GBM were indeed significantly different (*p* = 7.255e^−10^). Differently from *ADAR1*, both *ADAR2* and *ADAR3* were found strongly downregulated in GBMs (*p* ≤ 0.0001, Fig. 4a). Notably, the *ADAR* expression pattern as found in de novo GBM of TCGA was also found in a different and independent de novo GBM patient cohort (CGGA) (Additional file 2: Figure S1b).

Next, we asked whether there could be a correlation between *ADAR* expression (FPKMs) and editing level in terms of AEI, nREI, and REI indexes. According to both the Spearman and the Pearson tests, we found a positive correlation in both cerebral cortex (i.e., Pearson *r* = 0.39, *p* < 0.0001) and GBMs (i.e., Pearson *r* = 0.23, *p* = 0.004) only when we associated AEI and *ADAR1* (Fig. 4b). In the normal cortex, the recoding editing index (REI) positively correlated (i.e., Pearson *r* = 0.28, *p* < 0.0001) with *ADAR2*, while it is inversely associated with *ADAR3* (i.e., Pearson *r* = − 0.26, *p* = 0.0025) (Fig. 4b, TCGA). The above results were also recapitulated in the CGGA GBM dataset (Additional file 2: Figure S5). These findings are intriguing as they suggested that editing, within *Alus*, reproduces the *ADAR1* level, while editing at recoding sites directly correlates with *ADAR2* and is negatively influenced by *ADAR3*.

We then looked for possible associations between *ADAR* expression and patients’ clinical data, such as age, gender, and OS, by applying both Spearman’s and Pearson’s correlation tests. We report that both *ADAR1* and *ADAR2* negatively correlated with age in healthy people, with the *ADARs* being less expressed in elderly subjects (Fig. 4c, d); however, we found that *ADAR3* displayed an opposite trend by being over-expressed in older subjects (Fig. 4e). Most interestingly, when we evaluated whether these correlations were gender-dependent, we found that *ADAR3* positively and significantly correlated only when male patients were considered (Fig. 4f and Additional file 2: Figure S6). Of note, no gender-dependent differences were observed when *ADAR1/2* were considered (Additional file 2: Figure S6). Importantly, the correlations between *ADAR* expression and age/gender of patients were always lost when GBM samples were analyzed (Fig. 2c–f and Additional file 2: Figure S6).

In summary, we found that *ADAR* expression is decreased in GBM, matching with the overall editing reduction in this cancer compared to the normal brain, with *ADAR1* directly correlating with AEI and *ADAR2* directly associated with REI values. Interestingly, *ADARs* significantly associate with age/gender only in healthy individuals, while all these correlations were absent in GBMs.

### *ADAR3* is deleted in de novo GBM

**Fig. 5:**
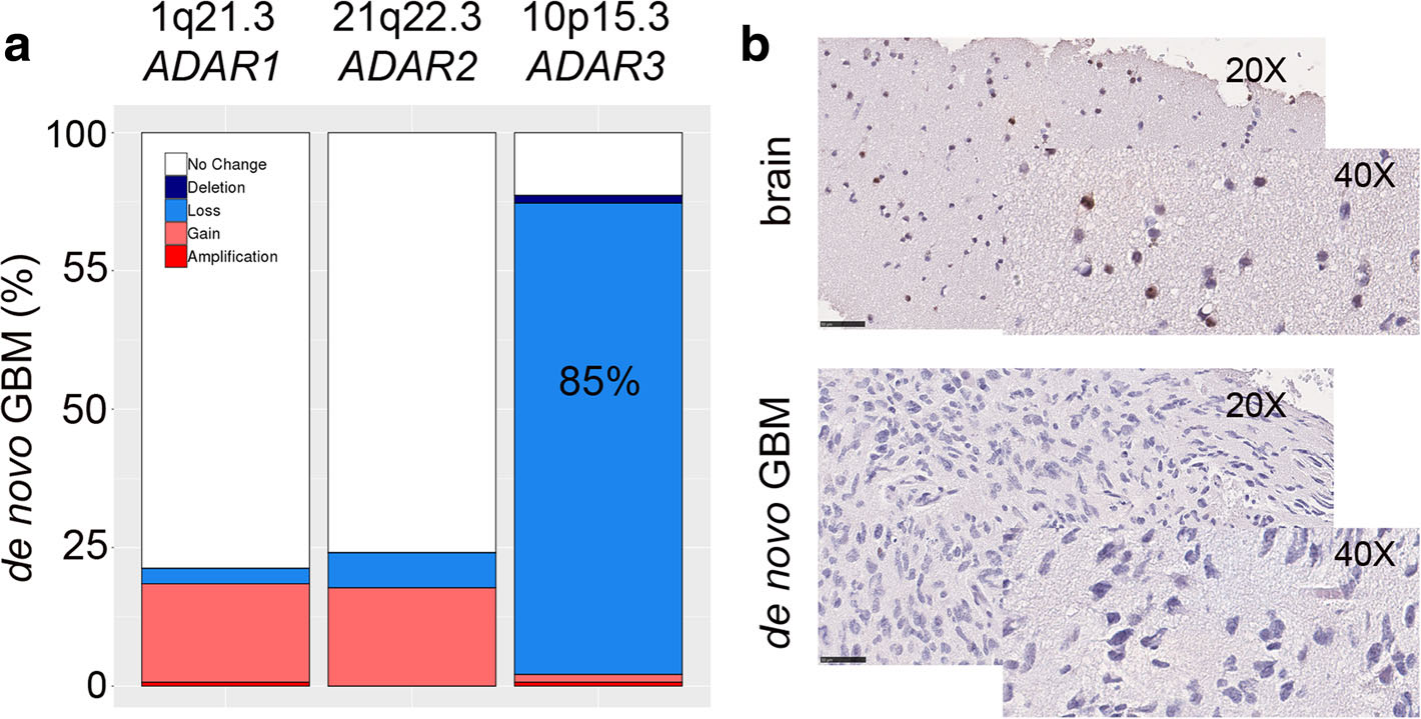
*ADARs* copy number variation in de novo GBMs. **a** *ADAR* genes copy number variation plot (cBioPortal, GISTIC2.0 algorithm). **b** Two representative pictures of six GBMs and two normal brains stained with ADAR3 by IHC (× 20 and × 40 magnifications are shown)

To investigate whether the decreased *ADAR* expression was due to DNA mutations or copy number variations (CNVs), we analyzed 145 GBM samples, included in our study, through the cBioPortal web tool with regard to variant calling Gistic2.0 CNV data generated by TCGA. No relevant mutations were observed within *ADAR* genes (data not shown); however, by analyzing for possible gene copy number variations, we found that > 85% of de novo GBMs tested carry an *ADAR3* deletion (with 85.1% hemizygous and 1.41% homozygous deletions) at the 10p15.3 genomic locus (Fig. 5a).

We validated these findings through qRT-PCR (data not shown) and by IHC performed on the normal brain and adult GBMs (Fig. 5b). Specifically, ADAR3 was found expressed in neuronal and glial cells (i.e., thalamus) while it was totally absent (0% positive cells) in the GBM tissues analyzed (Fig. 5b).

Overall, the downregulation of *ADAR3* as found in de novo GBMs is mainly accounted to a gene copy number alteration (deletion) in cancer.

### AEI associated with GBM patient’s overall survival in a gender-specific manner

Considering the important overall impact of RNA editing on cancer [15, 26–28], we asked whether inosinome might correlate with patients’ clinical parameters such as OS, age at diagnosis, or the Karnofsky Performance Score (KPS).

**Fig. 6:**
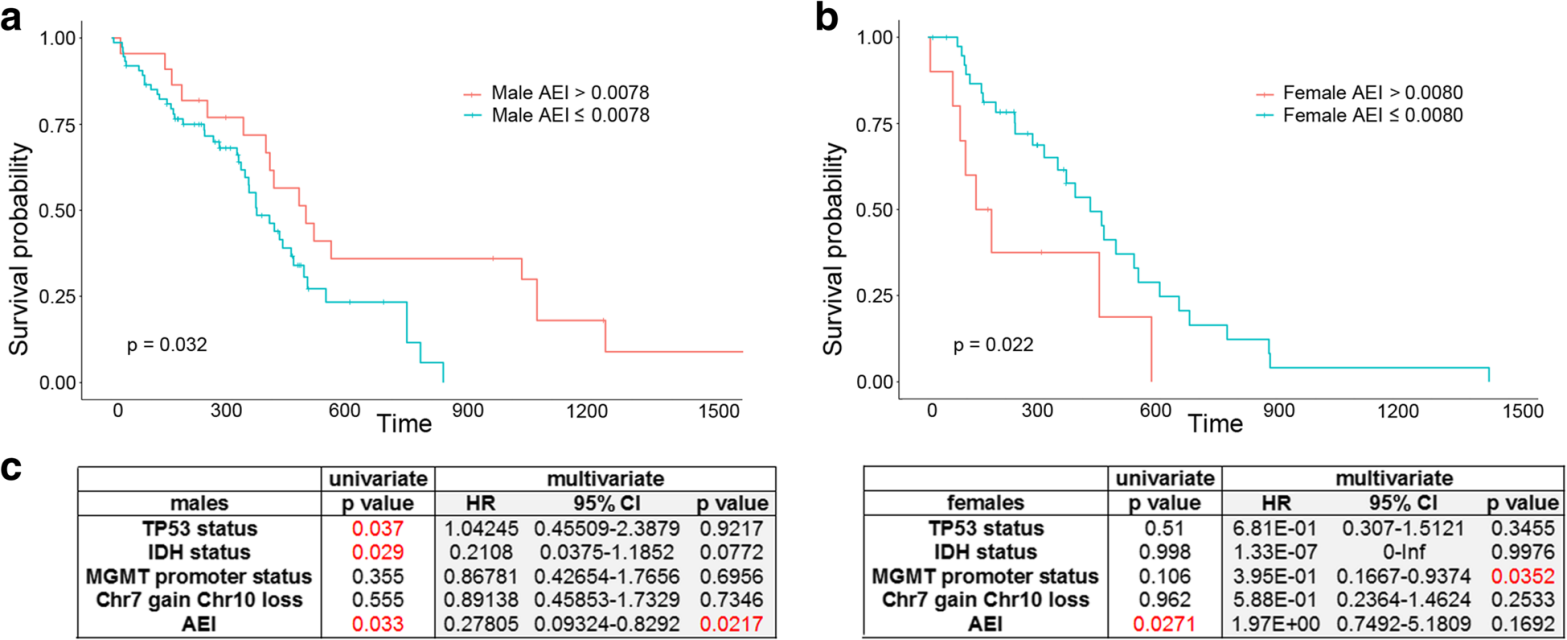
AEI associated with GBM patients overall survival in a gender-specific manner. **a**, **b** Gender-dependent association between *Alu* editing and OS in male and female GBM patients. Females and males exhibit an opposite trend when stratified using AEI. **c** Prognostic factors associated with OS in the Cox hazard regression analysis for de novo GBM patients from TCGA, univariate, and multivariate analyses are shown for male (left) and female (right), respectively

Firstly, we investigated whether the AEI, REI, and nREI indexes may represent predictive indexes for the patient’s outcome. Kaplan-Meier curves demonstrated that a significant correlation existed between AEI and patients’ OS with an opposite trend in females and males: a high AEI index (AEI > 0.0078, lines in red) correlated with a good prognosis only in males, while a lower Alu index (AEI ≤ 0.0078, lines in blue) was predictive of poorer outcome (Fig. 6a). In females, on the other hand, a lower *Alu* editing index (AEI ≤ 0.0080, lines in blue) is a reliable favorable prognostic factor associated with a better OS (Fig. 6b). The different gender-dependent trends of the KMs generated by AEI stratification were also observed in an independent RNA-Seq data of de novo GBM cohort (CGGA) (Additional file 2: Figure S7).

Next, univariate and multivariate tests were performed to estimate the independent effect of AEI with other factors predicted to be important in the OS of glioblastoma patients (males and females). We showed that factors, including *TP53* status, *IDH1/2* status, and AEI, were significantly associated with the OS of GBM male patients, whereas just the AEI came out as the only factor to be linked to the OS in female patients (Fig. 6c). Multivariate Cox regression analysis, which investigates how factors such as *TP53* mutations, *IDH1/2* status, *MGMT* promoter methylation, Chr7 Gain/Chr10 loss, and AEI jointly affected survival, was also performed. Our analysis indicated that AEI was an independent prognostic factor for GBM OS in male patients, demonstrating that patients with a high AEI have a significantly decreased risk of death (HR, 0.278; *p* = 0.0217) (Fig. 6c).

Overall, our data demonstrated that a high *Alu* editing index (AEI) positively correlates with a better patient outcome (OS) in males; however, particular attention is necessary for female GBM patients that survive less with a high AEI.

### Editing signature did not match with the TCGA-proposed GBM subclassification

**Fig. 7:**
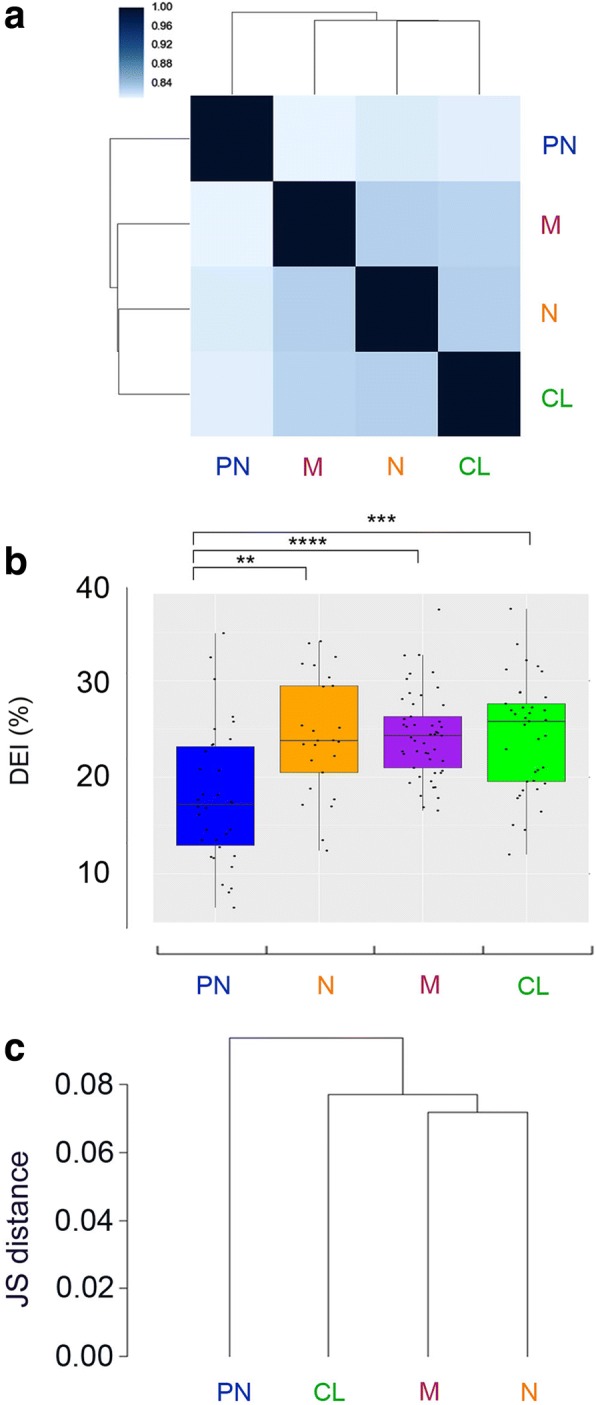
Inosinome signature did not overlay the previously proposed GBM subclassification. **a** Heatmap of clustered correlation matrix based on Spearman’s coefficients considering all the editing positions detected in proneural (PN, shown in blue), mesenchymal (M, shown in purple), neural (N, shown in orange), and classical (CL, shown in green) GBM subtypes. **b** Distributions of editing index (DEI) calculated at the differentially edited sites identified among GBM subtypes (see Additional file 4: Table S3). **c** Dendrogram plot representing Jensen-Shannon distances based on gene expression levels (cummeRbund software). ****p ≤ 0.0001, ***p ≤ 0.001, **p ≤ 0.01

GBMs were stratified into different subtypes based upon patterns of genomic mutations and gene expression [29, 30]. In order to see whether genetic and epigenetic events (such as RNA editing) might coincide, we investigated if RNA editing signature can overlap with the well-known glioblastoma subtypes (i.e., classical (CL), mesenchymal (M), neural (N), and proneural (PN) [29]). A hierarchical clustering based on Spearman’s correlation coefficients was applied and calculated by pairwise comparisons of RNA editing levels among GBM subtypes (CL, M, N, PN). Clustering was performed considering all the editing positions together. Interestingly, proneural GBMs are clustered as a different group to the others (Fig. 7a). In order to identify the sites responsible for the inosinome-based proneural separation, we performed all the possible site-by-site pairwise comparisons of editing levels among GBM subtypes focusing on non-repetitive sites and selected those that resulted significant (two-tailed Mann-Whitney *U* test) in at least 1 comparison. We identified 16 positions in 9 different genes (Additional file 4: Table S3 and Additional file 2: Figure S8). These sites were then used to calculate an ad hoc index, herein identified as “differential editing index” (DEI), that shows editing differences among known GBM subgroups (Fig. 7b, two-tailed Mann-Whitney *U* test). We also analyzed editing indexes (AEI, nREI, REI) *per* subtype; however, no significant differences were observed when using these indexes according to a two-tailed Mann-Whitney *U* test (data not shown).

GBM subtypes were also analyzed taking into account the gene expression signatures by means of Jensen-Shannon distance based on all the differently expressed genes. Once more, two distinct gene expression profiles were identified: one enriched with proneural subtype and the other including the three remaining subtypes (Fig. 7c).

Overall, our data showed that GBM inosinome signature did not correspond with GBM subclassification based on gene expression and mutational analysis (CL, M, N, PN), thereby suggesting that GBM is a highly multifaceted disease in which several layers of molecular information (including genetic and epigenetic) should be considered.

### Inosinome-based GBM patients stratification identifies a novel gender-dependent high-risk patient subgroup

**Fig. 8:**
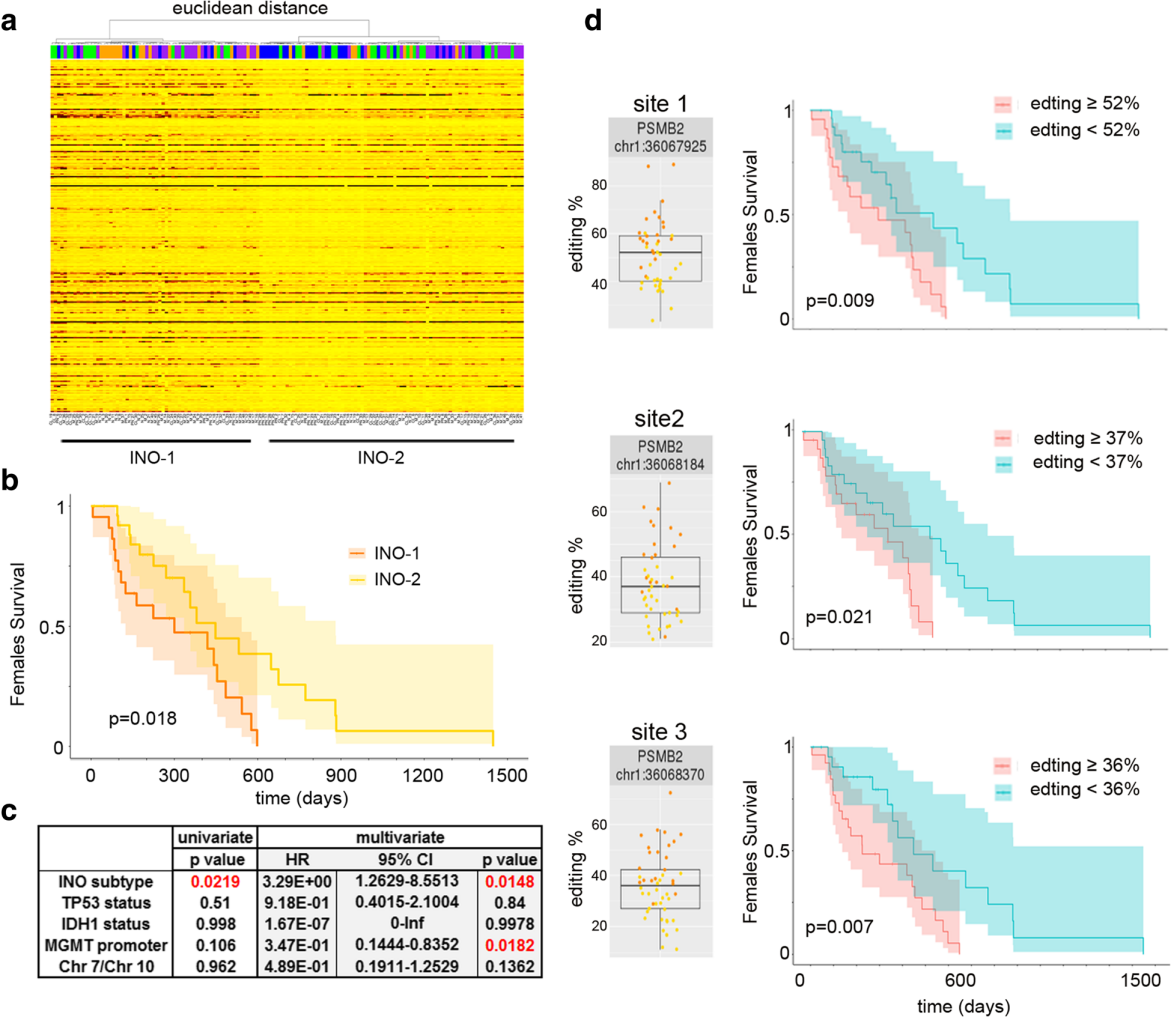
GBM patient stratification based on inosinome signature identifies two subgroups. **a** Unsupervised hierarchical clustering (based on Euclidean samples distance matrix) and heatmap (editing levels) calculated on the editing positions detected in at least 140 GBMs. **b** Kaplan-Meier curves representing the OS of GBM female patients stratified by INO-1 and INO-2. **c** Prognostic factors associated with the OS of de novo GBM female patients (TCGA) are analyzed with the Cox hazard regression, both univariate and multivariate analysis are shown. **d** Editing frequency distributions within 3′-UTR of *PSMB2* in female GBM patients and Kaplan-Meier curves based on high/low editing at the *PSMB2* sites in female GBM patients

Rigorous editing analysis of de novo GBMs was conducted in order to stratify patients by their RNA editing signatures. We first collected all the editing positions being detected in at least 140 GBMs that were further selected for positions laying within exons, introns, 5′-3′-UTRs (Additional file 5: Table S4); we then used the retained positions to calculate an Euclidean sample distance matrix with the resulting unsupervised hierarchical clustering, showing that GBMs can be classified into 2 distinctive subgroups: the GBM-Inosinome-group 1 (here indicated as INO-1) characterized by a higher editing signature and the GBM-Inosinome-group 2 (here indicated as INO-2) with an overall lower editing level (Fig. 8a). Editing index (AEI, nREI, and REI) distributions in INO-1 and INO-2 GBMs were significantly different (AEI and nREI *p* = 0.0001; REI *p* = 0.01) (Additional file 2: Figure S9a).

Interestingly, INO-1 GBM female patients are characterized by a worse prognosis (OS) compared to the INO-2 female subgroup (*p* = 0.018) (Fig. 8b). No differences in OS were found when we analyzed only males or all the GBM patients together (data not shown). The new female GBM stratification (INO-1/2) was also observed by analyzing an independent de novo GBM cohort (CGGA) (Additional file 2: Figure S10).

Multivariate Cox regression analysis was also performed to investigate whether factors such as *TP53* mutations, *IDH1/2* status, *MGMT* promoter methylation, Chr7 Gain/Chr10 loss, and INO subgroup jointly affected survival. Our analysis indicated that the INO patient’s stratification is an important prognostic factor for GBM female patients (HR, 3.2E+ 00; *p* = 0.014) (Fig. 8c).

Among the top differently edited positions identified in female INO-1 and INO-2 (with at least 15% Δ median, Additional file 6: Table S5), there were three editing positions all located within the same transcript identified as proteasome subunit beta type 2 (*PSMB2*). Interestingly, these three *Alu* sites are located within the 3′-UTR of *PSMB2* and were all significantly associated with patients’ outcome in females only, with higher editing correlating with a negative prognosis in these patients (Fig. 8d). We found a significant and direct correlation between *PSMB2* expression and editing only when female patients were considered (Additional file 2: Figure S11).

**Fig. 9:**
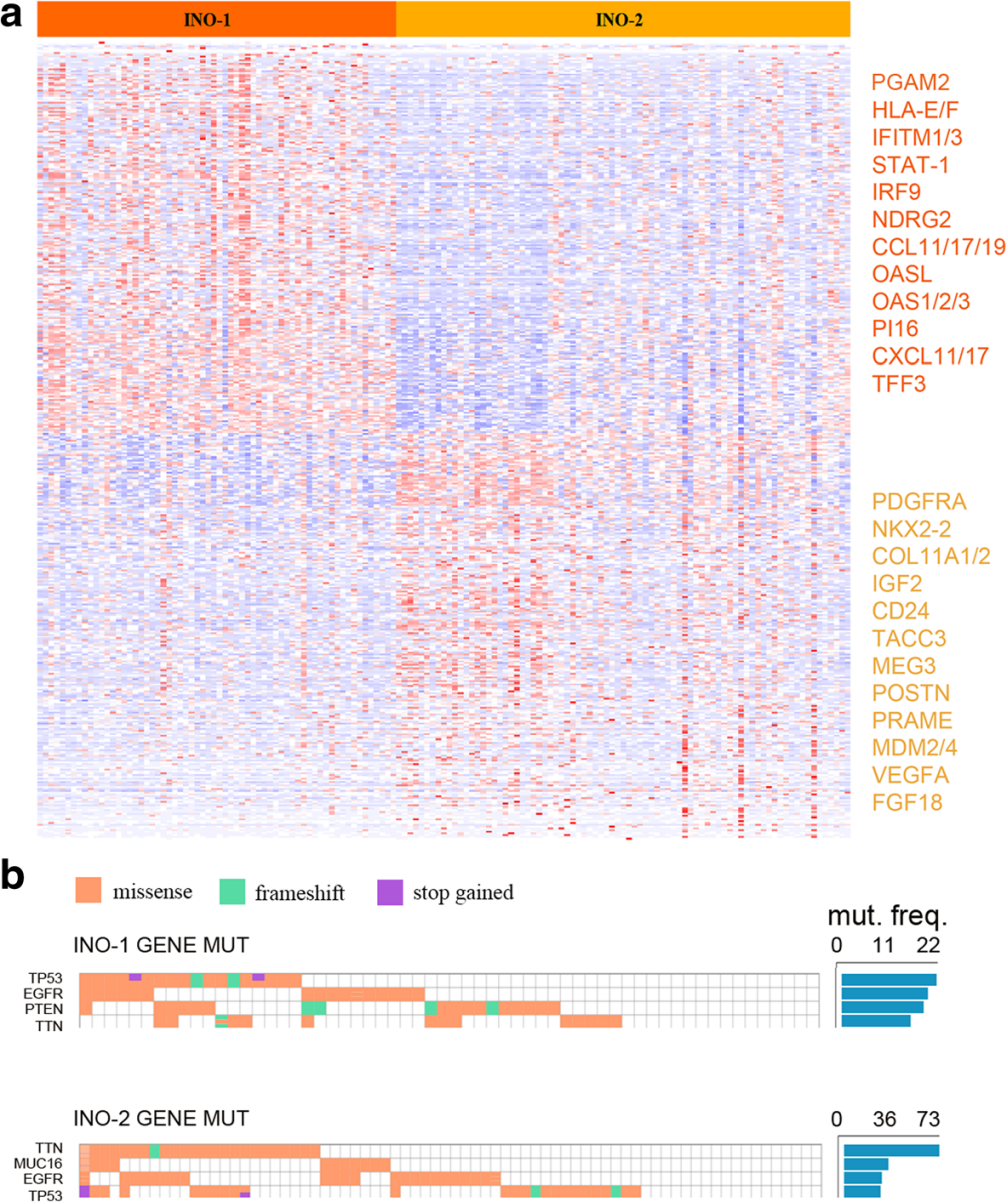
Gene expression profiles of INO-1 and INO-2 subtypes. **a** Heatmap showing the top differentially expressed genes between INO-1 and INO-2 GBM subtypes (Additional file 7: Table S6) in dark yellow listed some highly expressed genes in INO-1 and in light yellow the highly expressed genes in INO-2. **b** Mutational analysis of the most frequently altered genes in INO-1 and INO-2 GBM subgroups

The editing-based molecular classification proposed (INO-1 and INO-2) was further analyzed by gene expression and mutational signature (Fig. 9 and Additional file 7: Table S6). We found that INO-1 GBMs shows upregulation of genes mainly involved in metabolic pathways such as the D-amino acid oxidase (*DAO*), the phosphoglycerate mutase 2 (*PGAM2*), and the indoleamine 2,3-dioxygenase 1 (*IDO1*) as well as genes of immunological signature, such as the signal transducer and activator of transcription 1 (*STAT1*) or the interferon regulatory factor 9 (*IRF9*) (Fig. 9 and Additional file 7: Table S6). Of note, both *STAT1* and *IRF9* can upregulate *ADAR1* [31], and indeed, patients laying in INO-1 subgroup, characterized by a high expression of *STAT1* and *IRF9*, also showed an increased level of *ADAR1* (Additional file 2: Figure S9b).

INO-2 GBM subgroup is characterized by genes belonging to the p53-pathway (such as *MDM2/4* and *CCND1*) and by the upregulation of important genes such as *PDGFRA*, *IGF2*, and *VEGFA* and genes coding for nucleosome proteins (Fig. 9 and Additional file 7: Table S6).

Mutational profiles of GBM-INO-1 and GBM-INO-2 showed that GBMs with the majority of the *TP53* and *EGFR* mutations belonged to the INO-2 subgroup, in which we also reported higher presence (> 70% GBMs) of mutations within *TTN* gene. Of note, the analysis of differently expressed genes in INO-1 and INO-2 females showed that INO-1 patients have a high level of IFITM1, IDO1, and EGFR (Additional file 7: Table S6).

In summary, we described an inosinome-based molecular stratification of GBM patients that identifies two different subgroups (based on editing, gene expression, and mutational analysis), i.e., INO-1 and INO-2, which could be clinically relevant for the identification of gender-dependent high-risk GBM patients.

## Discussion

Our study provides a transcriptome-wide characterization of RNA editing across de novo GBM samples and shows that A-to-I RNA editing and the enzymes mediating this modification are significantly altered in this aggressive brain cancer. By correlating the inosinome-based data, *ADAR* expression, and patient features/outcome, we depicted the GBM inosinome landscape that indicates RNA editing as an exciting field of investigation for discovering alternative mechanisms clinically relevant for GBM patients and important for the RNA editing machinery in the brain.

A clear separation between cancer and normal samples (either tissues or cells) was observed based on their distinctive editing profiles. The origin of such differences involved all the editing positions in the transcriptome: *Alus*, non-repetitive, and recoding sites. We generated novel metrics to obtain an unbiased and robust measure of editing frequencies at non-repetitive (nREI index) and recoding (REI index) sites that, together with the AEI (*Alu* editing), showed an overall marked reduction of editing frequency in GBMs compared to normal samples. Among the recoding sites, 178 positions were found differentially edited with a > 93% of sites found under-edited in GBMs, thus expanding, on a larger scale, what was previously observed at a few specific sites [32–34]. The *GRIK2* Q/R, Y/C, and *GABRA3* I/M sites emerged as sites with a remarkable editing decrease in GBM (with an editing drop ranging from − 82 to − 74% Δ medians) followed by *GLI1* R/G site (− 27% Δ medians), the latter playing a key role in modulating the Hedgehog (HH) signaling in cancer [35]. The *GRIA2* Q/R (− 14% Δ medians) site, being less edited in GBM, appeared quite “resistant” to a generalized editing decrease tendency. Despite the global editing loss observed in GBM, we found that a few recoding sites (12 sites) were over-edited in cancer, with the top sites being *CADPS* E/G (+ 17% Δ medians) and *COG3* I/V (+ 17% Δ median). Both of these sites were localized in transcripts conserved during evolution (PHAST conservation score from 46 species ~ 700/1000) and involved in neuro-trafficking and exocytosis pathways [36, 37].

A recent pan-cancer study identified an appreciable number of RNA editing sites with potential clinical implications (*AZIN1*, *COG3*, and *GRIA2*) [18]*.* Our study further expands this observation and provides evidence that the specific RNA editing event within the *COG3* I/V site, particularly enriched in malignant gliomas, is pro-tumoral in glioblastoma cells boosting both proliferation and migration. Previous studies on large-scale RNA editing analyses, mostly using TCGA data, demonstrated an overall increased editing activity mainly due to ADAR1 [14, 18, 19], due to 1q amplification (chromosome where *ADAR1* lays), and/or due to the cancer inflammation state (ADAR1 is an interferon-responsive enzyme) [19]. Herein, we demonstrated that in de novo GBM, *ADAR* levels were profoundly altered with a strong decrease of *ADAR2* and *ADAR3*, both of which are significantly connected (even if with opposite trends) with the REI (editing at recoding sites) at least in normal brain and *ADAR1* correlating with the loss of AEI. The genomic alteration involving *ADAR3* was pervasive in GBM (> 85% deletion), but further investigations are required to dissect the interplay of *ADAR3* genomic loss and RNA editing perturbation in this cancer. The Kaplan-Meier analysis demonstrated that *Alu* editing is a prognostic factor in GBM in a gender-dependent manner, with high AEI representing a positive prognostic factor for male patients (66.2% of the 145 GBM patients analyzed) but becoming a negative factor in female patients. This finding supported a previous study which also indicated AEI as an important prognostic factor although in different cancer types [14].

Most importantly, we assessed the independent value of AEI with other variables known to be important for the OS of glioblastoma patients. Factors including *TP53* status, *MGMT* promoter status, Chr7gain/Chr10loss, and *IDH1/2* status were associated with the OS of GBM patients; interestingly, a Cox regression analysis including the above factors plus AEI showed that the editing index is indeed an independent prognostic factor for GBM OS in male patients, thereby demonstrating that male patients with a high AEI have a significantly decreased risk of death (HR, 0.278; *p* = 0.0217).

Intriguingly, based on the inosinome signature, we showed that de novo GBM patients can be stratified into two novel major subgroups: the GBM-Inosinome-group 1 (herein identified as INO-1 subgroup) including GBMs with a higher editing profiles and the GBM-Inosinome-group 2 (INO-2 subgroup) enriched of GBMs with a low editing signature at specific sites. The proposed novel INO-1 and INO-2 GBM subgroups also have distinctive gene expression profiles and mutational schemes; upregulation of genes such as *PGAM2*, *IDO2*, *STAT-1*, *IRF9*, *ADAR1*, and *HLA-E/A* in INO-1; and upregulation of *PDGFRA*, *IGF2*, *VEGFA*, *FGF18*, and *MDM2/4* in INO-2, the latter also showing a high frequency of mutations in *TTN*, *MUC16*, *EGFR*, and *TP53*. Importantly, female patients belonging to INO-1 have a bad prognosis (OS) compared to the INO-2 female subgroup (*p* = 0.018).

We showed that the INO subtype is a prognostic factor for GBM female patients (HR, 3.2E+ 00; *p* = 0.014) in a multivariate Cox regression analysis when *TP53* mutations, *IDH1/2* status, *MGMT* promoter methylation, Chr7 Gain/Chr10 loss, and INO subtype were jointly analyzed.

The intriguing gender-dependent editing signature, already found when AEI-KM was analyzed and re-observed in the INO1/2 stratification, opens new insights into RNA editing and cancer and also indicated that can be essential for the identification of gender-dependent high-risk patients.

Overall, female patients with low AEI (≤ 0.008049) or lying within the INO-2 subgroup have a better outcome (see Fig. 6b and Fig. 8b). Interestingly, both these groups of female patients showed a similar molecular signature characterized by a high mutation frequency rate (with *TTN* and *MUC16* being the top mutated genes) and an enrichment in the LGm6 DNA methylation profile [38] (Additional file 2: Figure S12).

INO-1 female patients (high-risk patients) showed a significantly higher editing level compared to the INO-2 group at specific positions (Additional file 6: Table S5). Among the top editing sites (with at least 15% Δ medians editing differences), we found three positions all lying within the 3′-UTR (within *Alus*) of *PSMB2*. The *PSMB2* transcript codes for the proteasome subunit beta type 2 and is one of the essential subunits of the proteasome. Proteolysis plays a major role in cancer cells, for example, controlling the degradation of transcription factors, such as p53, c-Jun, NF-kB, HIF-1a, STAT3, androgen receptors, and sterol-regulated element-binding proteins. Studies on proteasome inhibitors and screening based on small interfering RNA in GBM highlighted the potential significance of proteasome in cancer [39–41]. Interestingly, a high editing level at these three sites within 3′-UTR of *PSMB2* can potentially alter the binding of specific microRNAs leading to *PSMB2* upregulation in GBM. Accordingly, with the above observation, we found a significant and direct correlation between *PSMB2* expression and editing only when female patients were considered (Additional file 2: Figure S11). Of note, microRNAs can play an important role in sexually dimorphic neurobiological systems [42], indicating an intriguing connection between editing within 3′-UTRs and microRNAs differently expressed in an hormone-dependent manner.

The intriguing link between RNA editing and gender-related patient OS may suggest a possible contribution of gender-specific transcription factors or sex-dependent hormones controlling *ADAR* expression/activity at least in cancer. Previous work showed that editing in *fly* has some sex-specific differences within the central nervous system [43]. Indeed, in *fly*, the authors reported an overall reduction of editing in female relative to male heads as observed in *fru* neurons [43], which indeed exhibit sexual dimorphism [44]. Interestingly our findings open the possibility that also in the human brain there are gender-dependent differences in the overall impact of editing within specific sites and/or transcripts (i.e., *PSMB2*).

Another intriguing result of our study is that in normal individuals, the expression of the active deaminases, *ADAR1* and *ADAR2*, inversely correlated with patient age, being less expressed in older individuals. Differently, *ADAR3* displayed the opposite trend, being overexpressed in elderly subjects. The latter finding is particularly fascinating because *ADAR3* expression correlated with age only when male individuals were considered. This link between *ADAR* expression and age/gender was lost in GBM patients. Of note, we reported that > 85% of de novo GBMs carries a deletion involving the *ADAR3* genomic locus, a finding that opens the intriguing possibility that this specific event could play a possible role in GBM. A recent study reported *ADAR3* overexpression in few adult GBMs [7]. Differently, we showed that *ADAR3* is down-expressed in adult GBM due to the deletion involving its genomic locus, with only really limited cancer samples (~ 2.1% of GBMs) carrying a gene gain/amplification. It is possible that genetic differences among GBM samples can explain the discrepancies in different studies.

An important contribution to GBM research was the identification of GBM subtypes defined on the basis of genomic mutations and expression signatures [29, 30]. When we superimpose the RNA editing signature to the well-known GBM subtypes (with mesenchymal, neural, classical, and proneural) [29], we found that only 2 groups emerged with the proneural subtype came out as an independent group and a second GBM group including all the remaining subgroups (mesenchymal, neural, classical). We identified 16 editing sites in 9 different transcripts, all significantly under-edited in PN. Most of these sites lay within 3′-UTRs (17/22), indicating that editing may be linked to gene expression regulation. Seven sites were localized within *MDM2* upstream the p53-pathway [45]; of note, *TP53* mutations and loss of heterozygosity were frequent events in proneural subtype [29].

Overall, our study defined a novel framework in which editing profiles can play multiple and essential roles in GBM being important for (i) possible novel diagnosis (using AEI, nREI, REI, and *ADAR3* expression), (ii) an alternative GBM patient stratification with only two editing-based subgroups (INO-1 and INO-2), (iii) novel clinical prognostic factors for GBM patients (i.e., editing within *COG3* I/V and AEI), (iv) eligible target (*COG3* I/V) for personalized therapeutic intervention (i.e., antisense oligonucleotides), and (v) the detection of a novel high-risk gender-specific patient subgroups for which more aggressive treatments are necessary.

For many cancer types, men and women are very different in terms of susceptibility, survival, and mortality, and this is a fundamental issue for cancer prevention and therapy: however, little is known for GBM [46]. A recent study revealed that molecular differences in cancer between males and females determined strong sex effects due to mutations, DNA methylation, transcripts, and protein expression in several cancers but not in high-grade gliomas [47]. The present study shows, for the first time, that post-transcriptional events such as A-to-I RNA editing may play a key role in a gender-dependent GBM patients stratification that could be important for the identification of “ad hoc” sex-related therapies.

## Conclusion

Our study presents a comprehensive analysis of A-to-I RNA editing events in GBM, normal brain, and astrocytes and provides the first evidence that RNA editing plays an important role in patients’ outcome. We propose that RNA editing at specific recoding sites may act as a “driver” for tumor growth and that GBM inosinome can be considered for a novel patient stratification method providing a systematic molecular understanding of sex differences in GBM.

## Methods

### Processing of sequence reads

Brain normal controls (132 cerebral cortex), with 39 mean (37 median) millions of reads (paired ends), were downloaded from the Genotype-Tissue Expression (GTEx), and primary/de novo GBMs (145 samples), with 48 mean (50 median) millions of reads (paired ends), were downloaded from The Cancer Genome Atlas (TCGA). Both libraries, with the same read length (76 bp) and generated from polyaRNA, were downloaded upon authorization from the database of Genotypes and Phenotypes (dbGaP) with accession numbers phs000424.v7.p2 and phs000178.v10.p8, respectively.

Astrocyte dataset was downloaded from the NCBI Sequence Read Archive (SRA): SRP064454 study: RNA-Seq of healthy human astrocytes [48].

Additionally, for the data validation, we also downloaded 88 de novo GBMs from the Chinese Glioma Genome Atlas (CGGA) [24] (read length 101 bp; .fastq) generated from total RNA (SRP027383 and SRP091303).

TCGA GBM datasets were downloaded in .bam format and converted in standard .fastq using bam2fastq tool. GTEx datasets were downloaded in .sra format and converted in .fastq by means of fastq-dump program that is part of the SRA toolkit package.

### Quality control and alignment

Low-quality reads were discarded by filtering with the NGS QC Toolkit [49] and default parameters (cutoff read length for HQ = 70%, cutoff quality score = 20).

High quality cleaned reads were mapped against pre-indexed human genome GRCh37, transcriptome (pre-processed set of known splice junctions from Ensembl annotation), and dbSNP common release 144 using HISAT2 version 2.0.4. Unique and concordant alignments in .sam format were converted in the binary .bam format, sorted by genomic coordinates, and indexed by SAMtools.

For RNA-Seq experiments from normal astrocytes pools, duplicated reads were removed using the MarkDuplicates.jar tool from Picard package (https://broadinstitute.github.io/picard/).

### Detection of A-to-I editing at specific sites

RNA editing events were obtained merging known positions from RNA Editing ATLAS [10] (http://srv00.recas.ba.infn.it/editing/) repository and from RADAR version 2 database (http://rnaedit.com/) [50]. Both collections include A-to-I changes identified using rigorous computational pipelines. Merging ATLAS and RADAR positions yielded a comprehensive and non-redundant RNA editing catalog comprising 4.668.508 sites. The REDItools package (https://sourceforge.net/projects/reditools/) [51] was employed to call RNA editing events from this huge collection of positions. In particular, we used REDItoolDnaRna.py script to each .bam file by using the following parameters: -m 60,60 -q 30,30 -T ALLediting.sorted.gtf.gz -G ALLediting.sorted.gtf.gz -e -c 0,0 -n 0.0 -v 0 -p -u -l -z. REDItool tables were then parsed, and only edited positions supported by at least ten reads and at least with 1% editing were retained and used for downstream analyses. Editing sites that did not achieved the above cutoffs, in at least two tumor samples and two normal brains, were considered not informative and discarded.

### *Alu* editing index

*Alu* editing index (AEI), according to the methodology described in Bazak et al. [16], measures the averaged editing rates of adenosines in *Alu* elements, weighted by their relative expression levels. It may be quantified by the ratio of the number of A-to-G mismatches (presumably due to inosines) to the total number of reads nucleotides aligned to a genomic adenosine within an *Alu* repeat.

To calculate *Alu* editing index, we collected with REDItools all the mismatches between the aligned reads and the reference genome that occur within *Alu* elements (annotations for Alu genomic regions were downloaded from UCSC genome browser), discarding mismatches in read positions with quality Phred score < 30 and those located at sites reported as genomic SNPs in dbSNP (Common SNP build 144).

### Non-repetitive editing index—recoding editing index

In order to analyze the editing level in non-repetitive sequences and at the recoding sites, we introduced two new metrics, custom python script (provided as Additional file 8): the non-repetitive editing index (nREI) and the recoding editing index (REI). nREI and REI were evaluated for each normal brain, astrocyte, and GBM sample as the weighted average of the editing level in all the sites located in non-repetitive regions and recoding positions.

### Gene expression quantification

Transcriptome quantification at gene level was performed for each sample with Cuffquant [52], and differential expression was tested with CuffDiff2 software version 2.2.1 [53] (with --dispersion-method per-condition parameter). Reference human transcriptome was obtained from GENCODE Comprehensive gene annotation Release 26 back-mapped to GRCh37 (https://www.gencodegenes.org/). Annotations for rRNA genes were downloaded from UCSC genome browser selecting the RepeatMask table. Gene expression values are reported as fragments per kilobase of exon per million fragments mapped (FPKM). Transcriptome signature comparison was conducted by CummeRbund R package (http://compbio.mit.edu/cummeRbund/).

### Statistical analysis

Statistical analyses were performed using R with pandas and scipy modules in python. Non-metric multi-dimensional scaling (N-MDS) was carried out in R using the metaMDS function of the *vegan* package, providing as input a Spearman correlation matrix calculated from editing levels for each sample. Two-dimensional images depicting MDS clusters, heatmaps, and box plots were generated by ggplot2 package in R. Kaplan-Meier curves were inferred by using the R packages “*survival*,” ggfortify, and “*survminer*” with log-rank test, and the resulting plots were customized by applying the R package ggplot2, (https://ggplot2.tidyverse.org/).

Using the R packages “*survival*” and “*survminer*,” univariate and multivariate analyses were performed by applying the Cox proportional hazard model to assess the effect of several molecular risk factors (*TP53* status, *IDH* status, *MGMT* promoter status, Chr7 Gain/Chr10 loss, and *Alu* editing index) on patient OS.

### Editing-based glioblastoma reclustering

Two hundred sixty-seven editing sites detected in at least 140/145 GBM samples (and located in intronic, exonic, and UTRs regions) were selected and used to calculate a sample distance matrix with the *vegdist* function (method = “Euclidean”) included in the *vegan* R package; the resulting distance matrix was used to perform, with the hclust R function (method = “ward.D”); an unsupervised hierarchical clustering is shown as a dendrogram at the top of a heatmap generated with the heatmap.2 and ggplot2 R functions.

### Human tissues and cell lines

De novo GBM tumors and control brain tissues (adult subjects) were dissected and either immediately frozen (for molecular studies) or embedded in *paraffin* (for immunohistochemistry analysis).

Human GBM cell lines U87-MG, U118-MG, and A172 were obtained from the American Type Culture Collection (ATCC) and routinely maintained in Dulbecco’s modified Eagle’s medium (DMEM) supplemented with 10% fetal bovine serum (Gibco-Life Technologies), 100 U/ml penicillin, and 100 μg/ml streptomycin, at 37 °C in 5% CO_2_. Mycoplasma contamination was routinely tested by Venor®Gem OneStep Mycoplasma detection kit for standard PCR (Minerva Biolabs), and the latest analysis was performed on February 2018. The cells were utilized between passage numbers 10 and 15.

### Editing activity in cell line

HEK 293T cells were chosen for the assessment of EGFP-ADAR2 and EGFP-ADAR1 editing activity as the endogenous editing activity in this cell line is undetectable. Cells were transiently co-transfected independently with 4 μg of EGFP-ADAR2, EGFP-ADAR1, and pEGFP (V-EGFP) 48 h after transfection; total RNA was extracted from the cells using TRIzol reagent and reverse-transcribed by specific primers for edited substrates. Transfection was tested by both real-time PCR and Western blot.

### RNA isolation and reverse transcription

Total RNA was isolated with TRIzol reagent (Invitrogen) from de novo GBM tumors, control brain tissues (adult subjects), and cultured cell lines, according to the manufacturer’s instructions. The cDNA pools were generated by SuperScript II reverse transcriptase (Invitrogen) using random hexamer primers.

### Analysis of RNA editing

Direct sequencing was performed on cDNA pools, and editing was calculated as described previously [54], or the PCR products were subcloned into the T-easy vector (Promega), and ~ 50 individual cDNA clones were sequenced for each sample. A to G changes in the individual clones were analyzed. For each sample, 2–3 independent RT-PCR reactions were performed.

### GBM cell lines and lentiviral infection

Human GBM cell lines U87-MG, U118-MG, and A172 were obtained from American Type Culture Collection (ATCC) and routinely maintained in Dulbecco’s modified Eagle’s medium (DMEM) supplemented with 10% fetal bovine serum (Gibco-Life Technologies), 100 U/ml penicillin, and 100 μg/ml streptomycin, at 37 °C in 5% CO_2_. Mycoplasma contamination was routinely tested by Venor®Gem OneStep Mycoplasma detection kit for standard PCR (Minerva Biolabs). The cells were utilized between passage numbers 10 and 15. Lentiviral *COG3* expressing vector [ABM LV484109 (pLenti-GIII-CMV-GFP-2A-Puro)] was purchased from ABMGOOD, and site-directed mutagenesis was performed to generate a lentiviral vector encoding the *COG3* edited version containing an isoleucine-to-valine substitution at position 635 (I635V). The lentiviral particles were produced by transfecting human embryonic kidney 293T (HEK293T) cells with the *COG3* unedited or *COG3* edited I635V expression vectors and the Lentiviral Packaging Mix (pREV, pMDL, and VSV.G). The supernatant was collected after 72 h and concentrated by ultracentrifugation at 40,000 rpm for 75′ at 4 °C. Cells were infected, and stable cells were selected with 1 μg/mL puromycin.

### MTS colorimetric assay

Cell proliferation was measured daily by 3-(4,5-dimethylthiazol-2-yl)-5-(3-carboxymethoxyphenyl)-2-(4-sulfophenyl)-2H-tetrazolium inner salt (MTS) using CellTiter 96 AQueous One Solution Cell Proliferation Assay (Promega). Absorbance intensity was determined on a microplate reader at 490 nm. The assay was repeated three times in triplicate. For statistical analysis, we used the two-sided *t* test.

### Migration assay

Transwell inserts with 8 μm pore size in 24-well plates (Corning, Life Sciences) were used for migration assays. 1.5 × 10^4^ cells were added to the upper chamber in 0.2 ml serum-free medium; the bottom chamber contained medium with 10% FBS which acted as cell attractant. After 24-h incubation, cells that reached the underside of the filter were stained with Diff-Quik staining set (Medion Diagnostics) and counted based on five field digital images taken randomly at × 10 magnification. Three independent experiments were performed. For statistical analysis, we used the two-sided *t* test.

### Real-time qRT-PCR

Quantitative real-time PCR (qRT-PCR) was performed to validate the expression of *COG3* in infected cells using pre-designed stem-loop primers (TaqMan MicroRNA Assay, Applied Biosystems-Life Technologies). cDNA was synthesized from 1 μg of total RNA (pre-treated with DNase I) by the ImProm-II Reverse Transcription System (Promega) using random hexamer primers according to the manufacturer’s instructions.

*GAPDH* was used as a control for normalization of mature mRNAs. The relative amount of *COG3* was calculated by the 2^−ΔΔCT^ method. Expression levels were represented as a relative fold increase compared to the control sample, which was arbitrarily set to 1. All qRT-PCR reactions were performed in duplicates, and *p* values were calculated (two-sided *t* test). The primers were supplied by Applied Biosystems: *GAPDH*, ID Hs99999905_m1; *COG3* ID Mm00616765_m1.

### Immunohistochemistry

Formalin-fixed, paraffin-embedded sections (3 μm thick) were mounted on positively charged glass slides. Deparaffinization and antigen retrieval was performed using the PT link instrument (Dako) and the EnVisionTM FLEX, low pH solution (Dako). Endogenous peroxidase was blocked by hydrogen peroxide (Sigma), and then sections were incubated ON at 4 °C with mouse monoclonal antibody anti-ADAR3, (3.591): sc-73,410, 1:100 dilution (Santa Cruz Biotech) followed by EnVision FLEX/HRP (Dako). 3,3′ Diaminobenzidine was used as the enzyme substrate to observe the specific antibody localization, and Mayer hematoxylin was used as a nuclear counterstain.

## Additional files

Additional file 1 Table S1. Alu editing index (AEI), non-repetitive index (nREI), recording editing index (REI) values, and hyper-editing in GBMs, normal brain, and astrocytes. (XLSX 35 kb)

Additional file 2 Figure S1-S12 with figure legends. (PDF 1404 kb)

Additional file 3 Table S2. Median values and statistics of significantly modulated recoding editing sites (178 sites) in glioblastomas (GBM) compared to normal brain cortex (GTEx). (XLSX 29 kb)

Additional file 4 Table S3. Editing sites characterizing the GBM subtypes. On the first sheet are shown the identified editing positions and in the second one are indicated the differential editing index (DEI) values. (XLSX 20 kb)

Additional file 5 Table S4. Editing-based GBM reclustering. (XLSX 31 kb)

Additional file 6 Table S5. GBM reclustering editing-based INO-1 and INO-2 in female patients. (XLSX 29 kb)

Additional file 7 Tables S6. Gene expression analysis of INO-1 and INO-2 GBM subgroups. In the first sheet are reported values of all patients and in the second sheet are shown the female values. (XLSX 83 kb)

Additional file 8 Custom Python Script. Source code for the custom script used to calculate the nREI and REI metrics. (PY 1 kb)

## Abbreviations

AEI: *Alu* editing index
DEI: Differential editing index
dsRNA: Double-stranded RNA
FPKM: Fragments per kilobase of exon per million fragments mapped
GBM: Glioblastoma
KPS: Karnofsky Performance Score
N-MDS: Non-metric multi-dimensional scaling
nREI: Non-repetitive editing index
OS: Overall survival
REI: Recoding editing index
TCGA: The Cancer Genome Atlas
