# Supplementary material for: Dynamic inosinome profiles reveal novel patient stratification and gender-specific differences in glioblastoma
Source: Genome Biol. 2019 Feb 13;20:33. doi: 10.1186/s13059-019-1647-x (PMC6373152; doi:10.1186/s13059-019-1647-x)
Supplement: Supplementary file 2 — Figure S1-S12 with figure legends. (PDF 1404 kb) [file 13059_2019_1647_MOESM2_ESM.pdf]

Additional File 2: Figures S1-S12

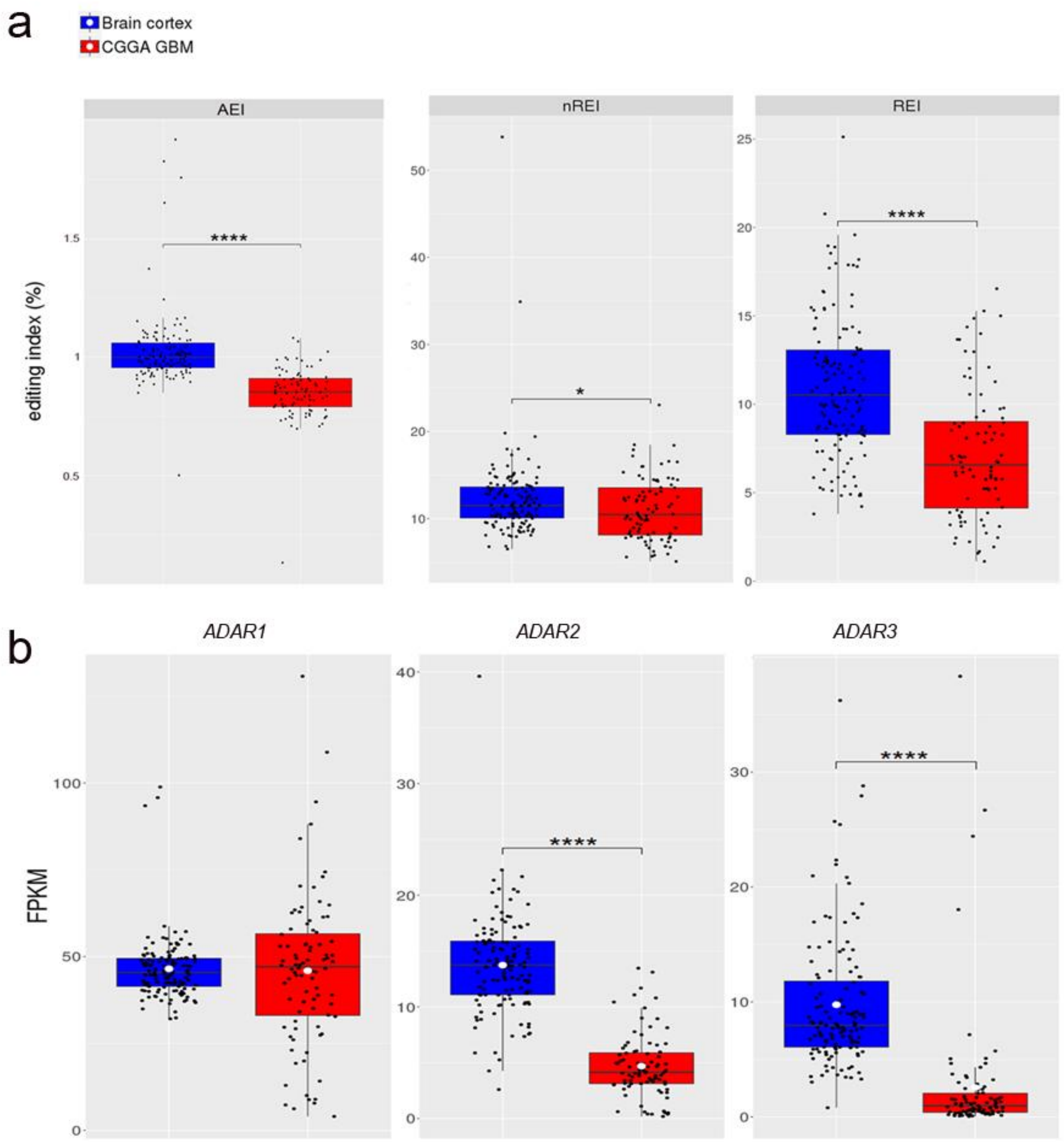

Figure S1. Inosinome signature (AEI nREI and REI) and ADARs expression in CGGA dataset. (a) *Alu* Editing Index (AEI), non-Repetitive Editing Index (nREI) and Recoding Editing Index (REI) values distributions (box plot, median) are shown. Two-tailed Mann-Whitney U test was applied. (b) *ADAR1*, *ADAR2* and *ADAR3* expression levels were calculated by using Cufflinks (FPKM distributions). * $p \leq 0.05$, ****$p \leq 0.0001$.

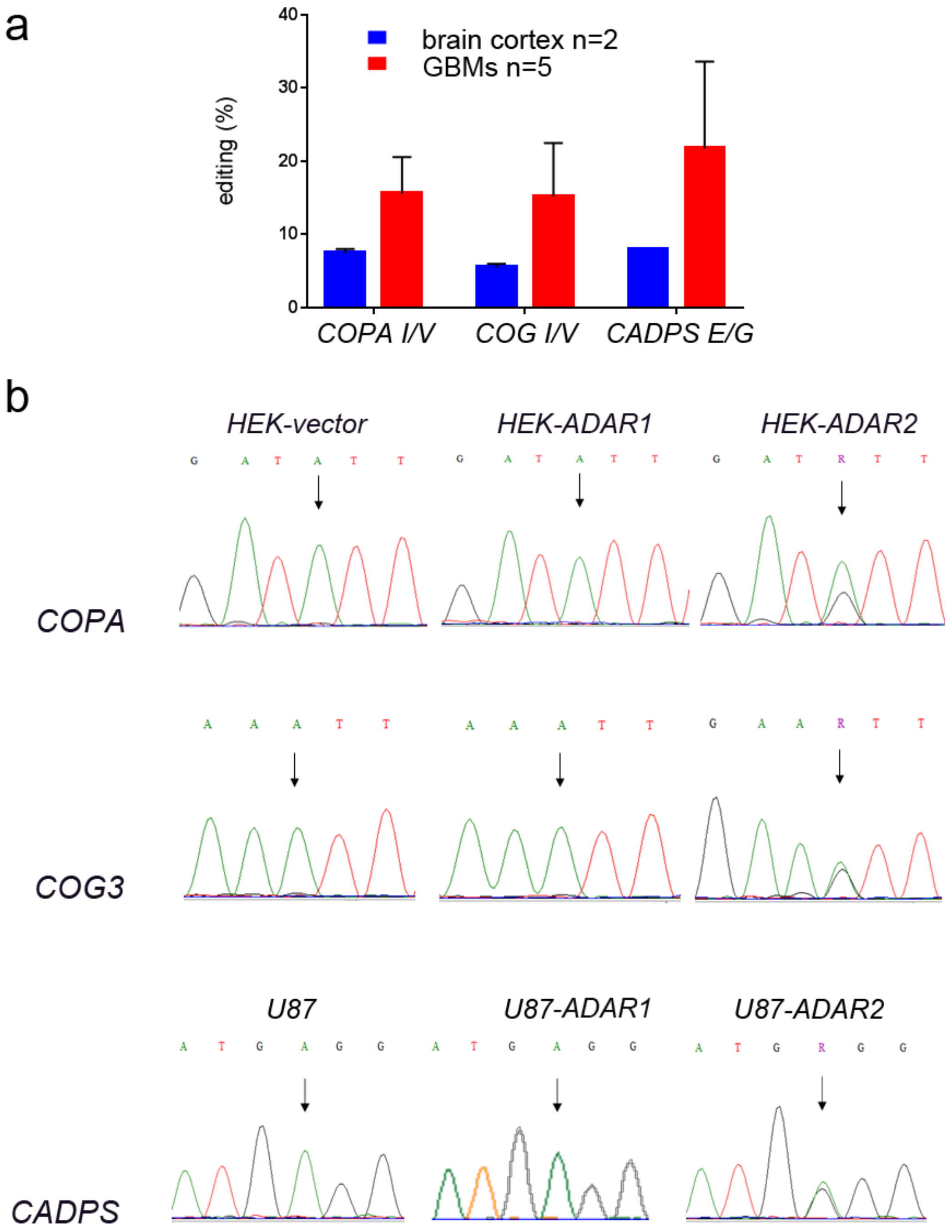

Figure S2. RNA editing at *COPA* I/V, *COG3* I/V, *CADPS* E/G sites. (a) RNA editing levels at *COPA* I/V, *COG3* I/V, *CADPS* E/G sites were tested in 5 different *de novo* GBMs and in 2 normal brain cortex, demonstrating that these sites were over-edited in GBMs compared to normal brain. (b) Chromatograms of *COPA* I/V, *COG3* I/V sites as tested in HEK293T cells in which we overexpressed at similar levels either *ADAR1* or *ADAR2* or the empty vector. The *CADPS* E/G site, not detectable in HEK293T cells, was analyzed in U87-MG glioblastoma cells in which we overexpressed *ADAR1*, *ADAR2* or the empty vector.

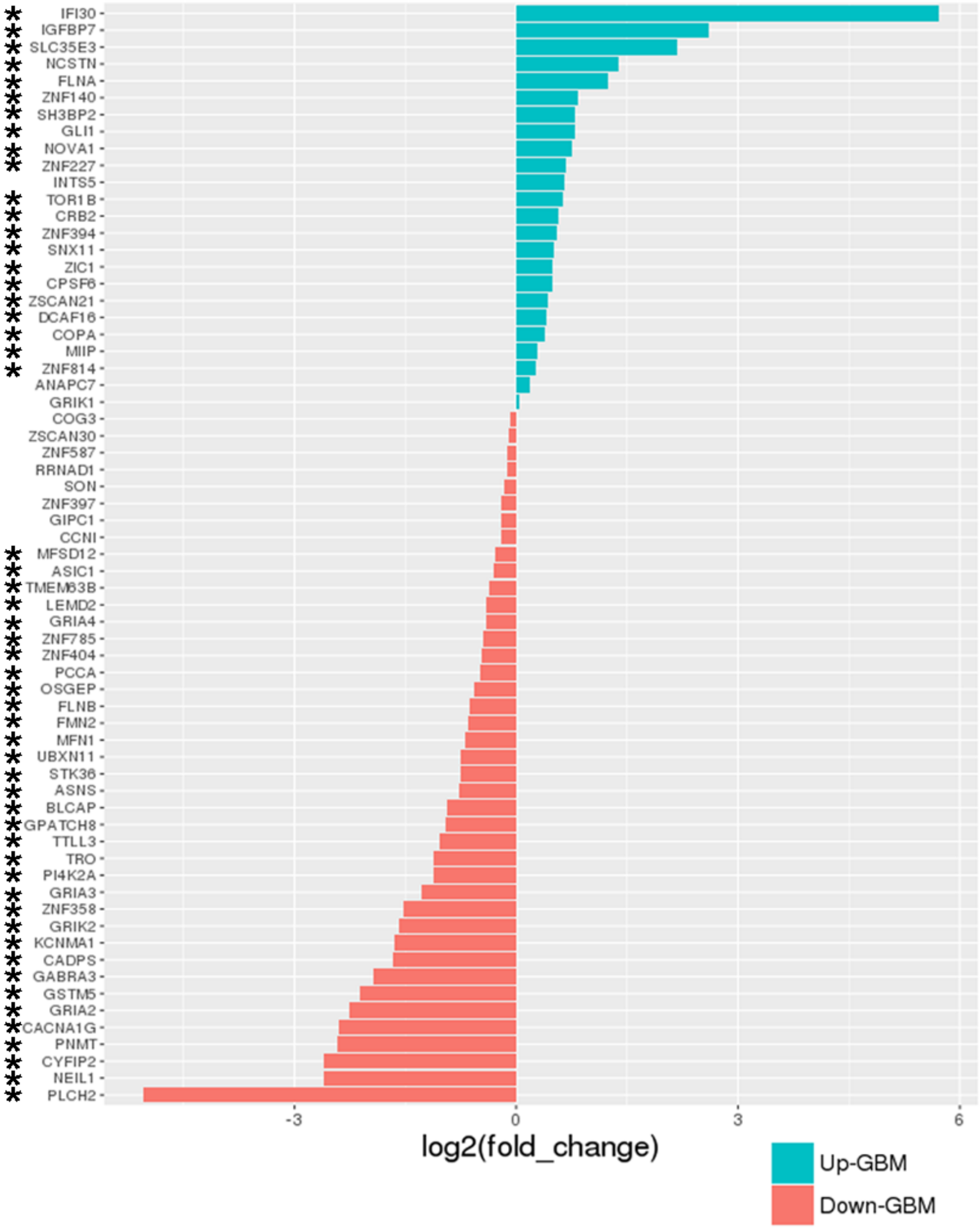

Figure S3. Expression levels of transcripts carrying recoding editing site/s. Expression levels of the 65 differentially edited mRNAs in *de novo* GBMs compared to the normal brain cortex reported as log2 (fold change) as calculated by Cuffdiff2. Asterisks indicate the significantly modulated transcripts ($q \leq 0.05$).

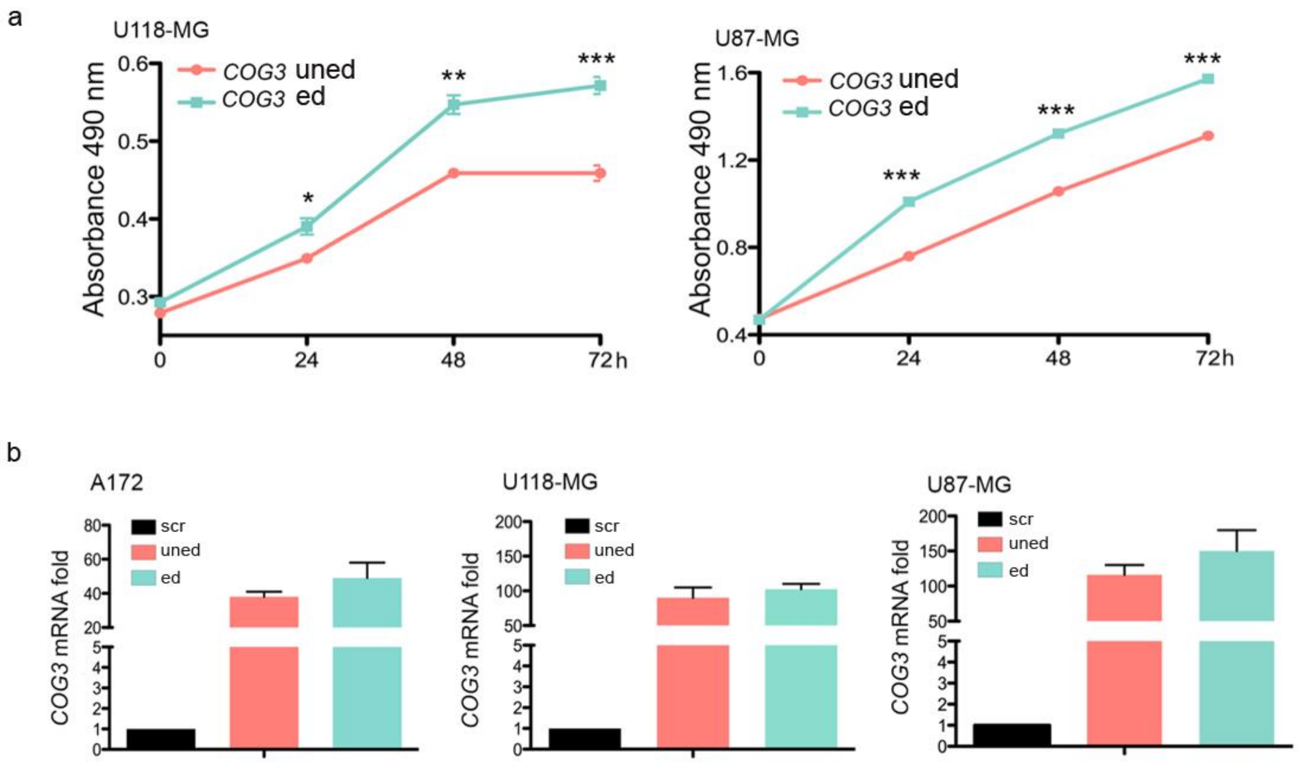

Figure S4. *COG3* I/V editing site has a pro-tumoral effect in GBM. (a) Cell proliferation of U118-MG and U87-MG glioblastoma cells infected with either unedited or edited *COG3* I/V. Mean ± sd (n=2) *p < 0.05, **p < 0.01 ***p < 0.001 (two-sided t-test). (b) Unedited COG3 and edited COG3 I/V expression (qRT-PCR) after infection. *COG3* expression levels were normalized to *GAPDH* and calculated as relative-fold increase compared to the scramble. Mean ± sd (n=3).

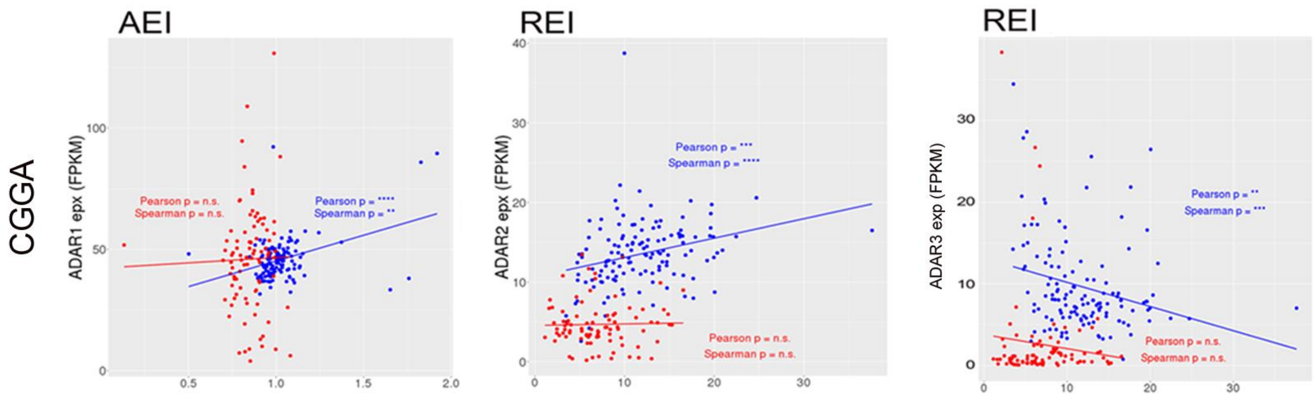

Figure S5. Correlations between *ADARs* expressions and Editing Indexes (AEI, nREI and REI) in CGGA GBM cohort and normal brain. Correlations (Pearson and Spearman tests) of *ADARs* expression (FPKM) and editing indexes (AEI, REI, nREI) are shown as calculated in normal brain (GTEx) and GBMs CGGA. $^{**}p \leq 0.01$, $^{***}p \leq 0.001$, $^{****}p \leq 0.0001$.

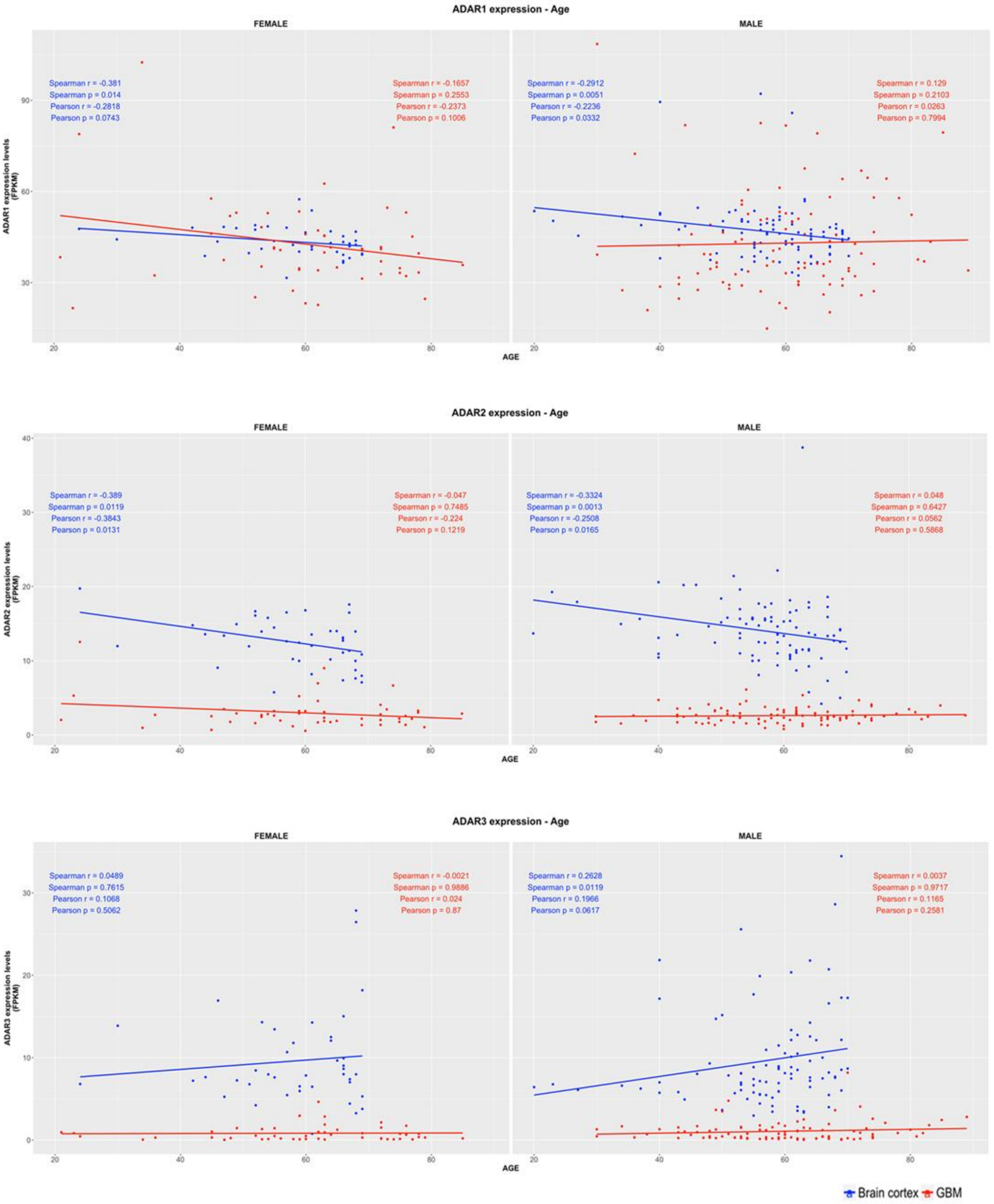

Figure S6. Correlation between *ADARs* expression and age. Correlation of age and *ADARs* expression in healthy people (indicated in blue) and GBM patients (indicated in Red). Pearson and Spearman correlation p-values are indicated.

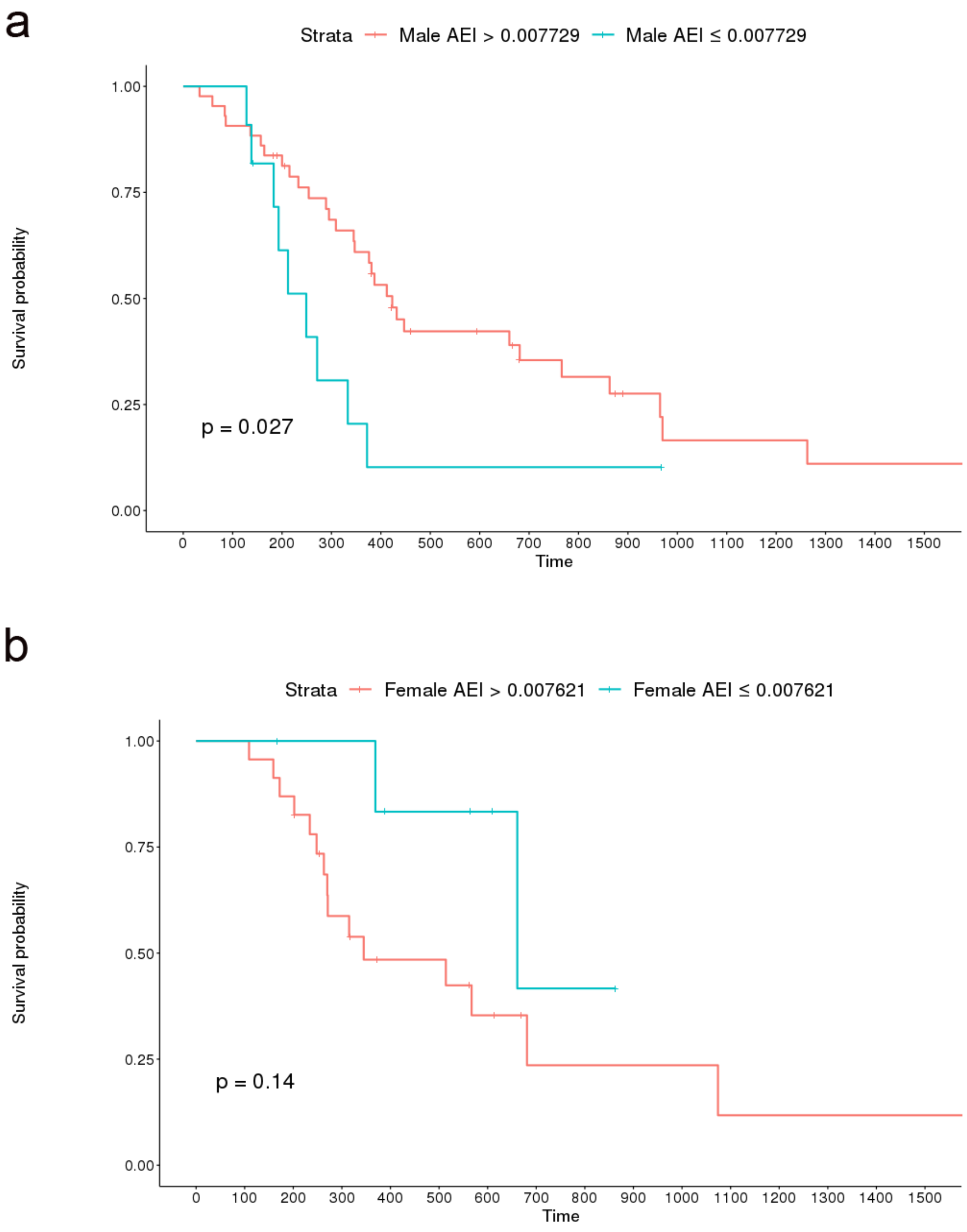

Figure S7. AEI associated with GBM patient overall survival in a gender-specific manner in the CGGA dataset. Kaplan-Meier analysis demonstrates a gender-dependent association between *Alu* editing and overall survival (OS) in males (a) and females (b) GBM patients. The optimal cutoff for AEI were chosen using the surv_cutpoint function included in the "*survminer*" R package.

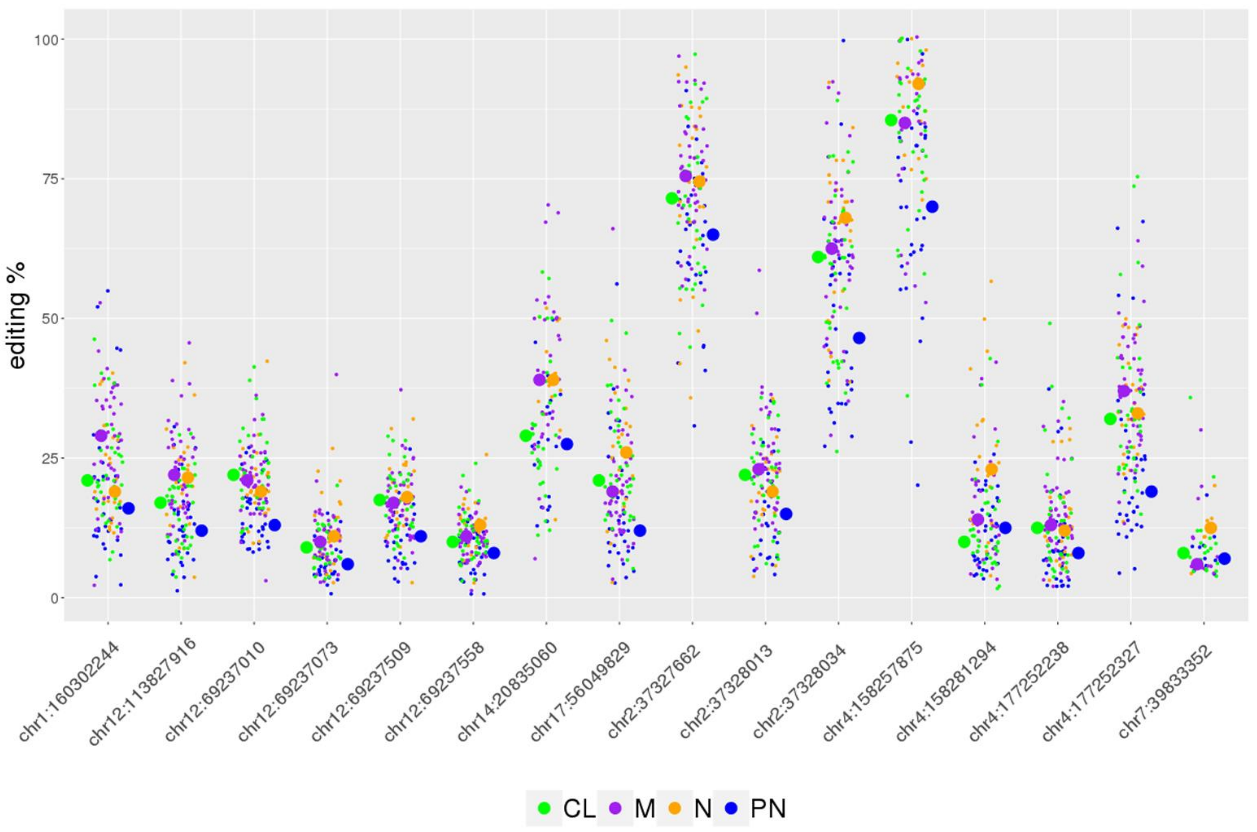

Figure S8. **Edited sites distribution among GBM-subtypes**. Scatter plot of editing values distributions among GBM-subtypes (M, CL, N, PN) were shown, larger dots represent medians values (Figure 7b, Table S3).

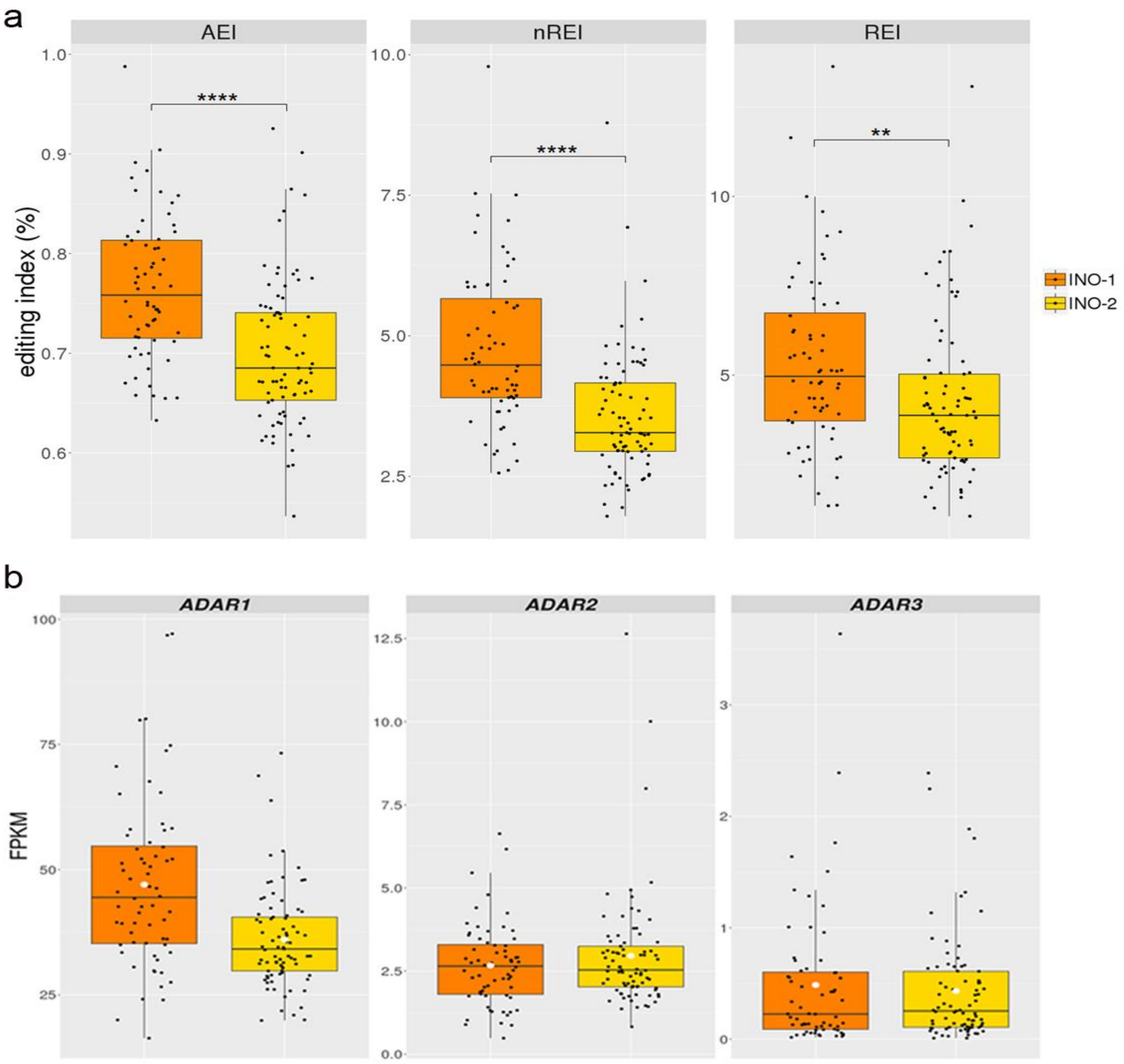

Figure S9. **Editing levels and *ADAR*s expression in INO-1 and INO-2 TCGA GBM patient's subgroups.** (a) Editing Indexes distributions (box plot, median) and (b) *ADARs* expression (FPKM, Cuffdiff2) in INO-1 (dark yellow) and INO-2 (light yellow). Black lines indicate medians and white dots indicate means. $**p \leq 0.01$, $****p \leq 0.0001$.

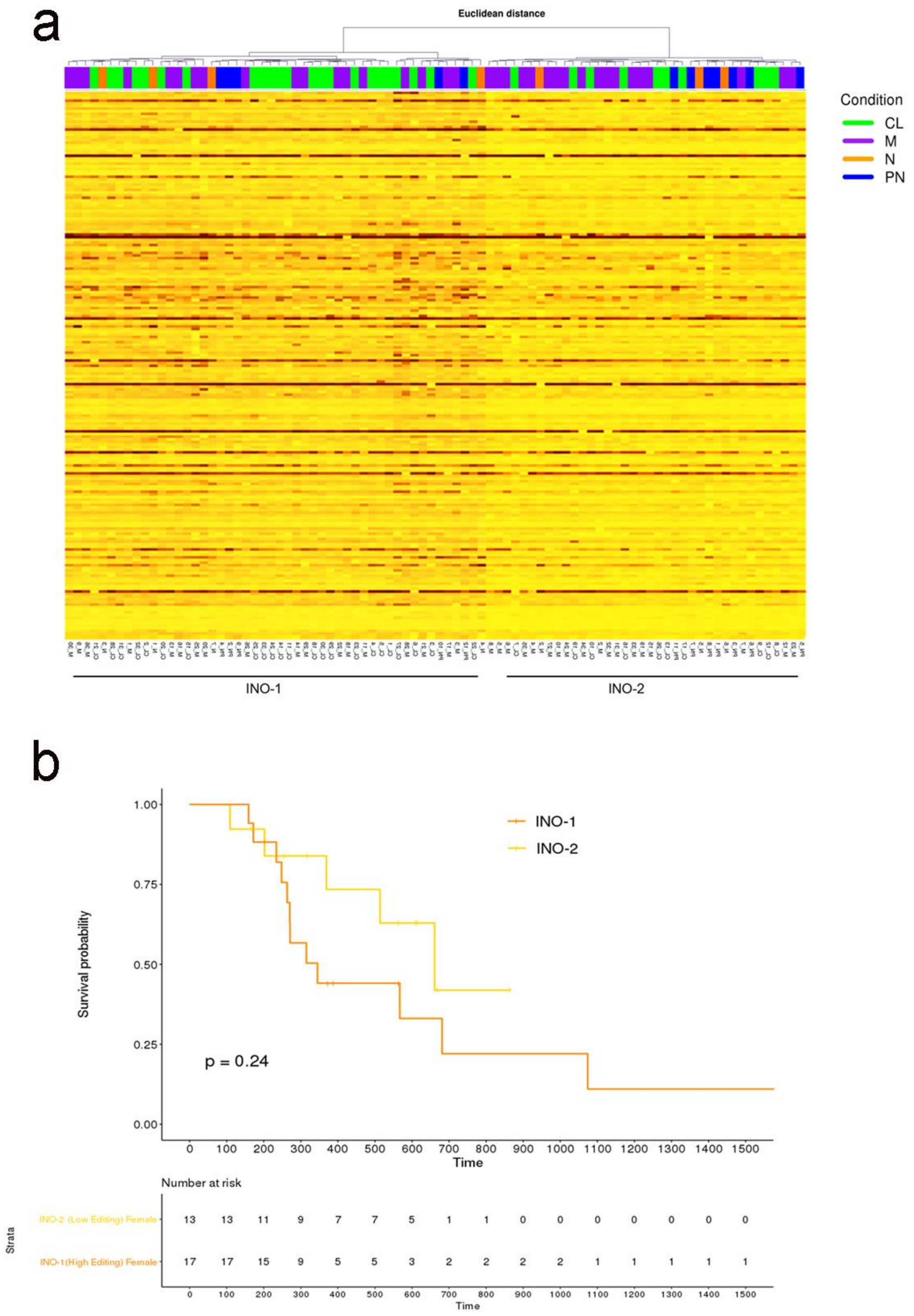

Figure S10. CGGA GBM patient stratification based on Inosinome signature identifies two subgroups (INO-1/2). (a) Unsupervised hierarchical clustering (based on Euclidean sample distance matrix) and heat map (editing levels). (b) Kaplan-Meier curves representing the OS of CGGA GBM female patients stratified by INO-1 and INO-2.

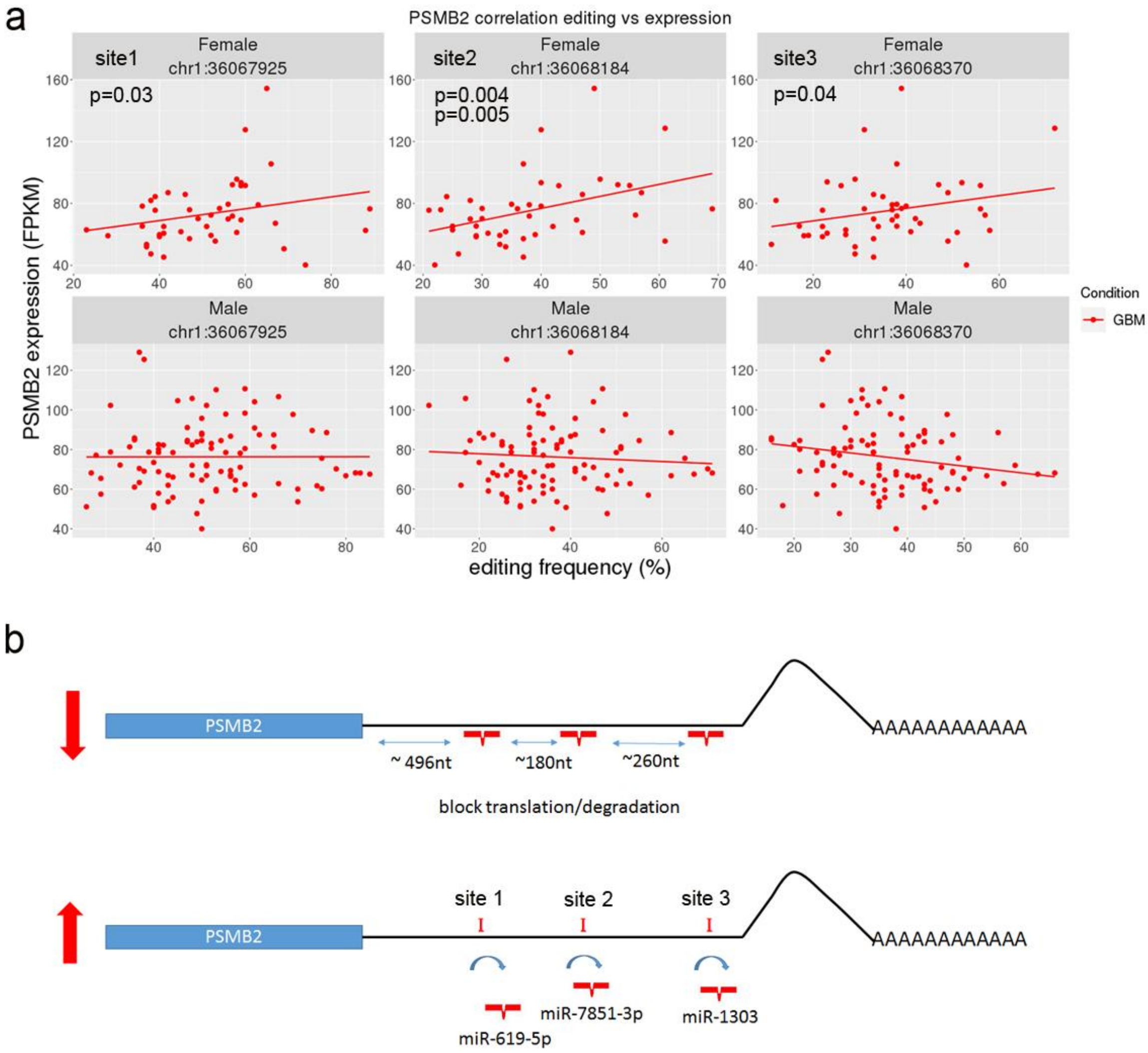

Figure S11. Editing at *PSMB2* sites correlates with *PSMB2* expression in female GBM patients. (a) Correlation between editing frequency (%) and *PSMB2* gene expression (FPKM) at three selected sites located in the 3'UTR region only in the female GBM patients. (b) The cartoon shows a hypothesized mechanism regulating *PSMB2* expression by editing within its 3'UTR. As example, we indicated some microRNAs (as identified by miRBase annotation, http://www.mirbase.org/) that can specifically bind the 3'UTR at the editing positions (Site 1, Site 2 and Site 3).

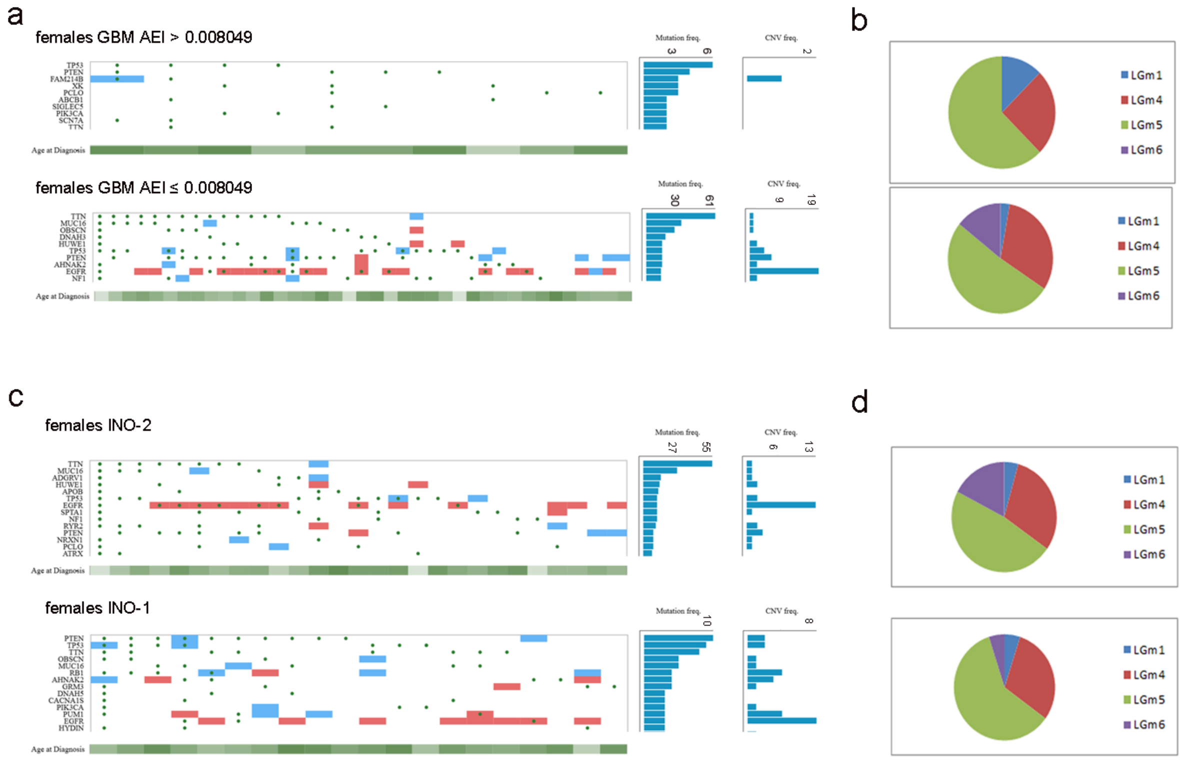

Figure S12. Molecular signature of GBM female patients. Female GBM patient's distribution (AEI cutoff 0.008049) and INO-1/2 were analysed for (a, c) mutations frequency, Copy Number Variations (CNV) and (b, d) methylation profiles distributions.
